# Integrated multi-omics reveals GABARAP-mediated mitophagy and pyruvate metabolism as key drivers of osteosarcoma progression

**DOI:** 10.3389/fimmu.2025.1680554

**Published:** 2025-11-19

**Authors:** Xiuxin Han, Yiqin Li, Yongheng Liu, Feng Wang, Tingfang Li, Qingchen Zhang, Guowen Wang, Jinyan Feng

**Affiliations:** 1 Department of Bone and Soft Tissue Tumors, Tianjin Medical University Cancer Institute and Hospital, Tianjin, China; 2 State Key Laboratory of Druggability Evaluation and Systematic Translational Medicine, National Clinical Research Center for Cancer, Tianjin, China; 3 Tianjin Key Laboratory of Cancer Prevention and Therapy, Tianjin’s Clinical Research Center for Cancer, Tianjin, China; 4 Department of Genetics, School of Basic Medical Sciences & The Province and Ministry Co-sponsored Collaborative Innovation Center for Medical Epigenetics, Geriatrics Institute General Hospital, School and Hospital of Stomatology, Tianjin Medical University, Tianjin, China; 5 Tianjin International Joint Academy of Biomedicine, Tianjin, China; 6 College of Pharmacy, Nankai University, Tianjin, China

**Keywords:** Osteosarcoma, Single-cell RNA-sequencing, mitophagy, pyruvate metabolism, GABARAP

## Abstract

**Background:**

Osteosarcoma is a highly aggressive bone malignancy characterized by frequent metastasis and therapy resistance. Although mitophagy and pyruvate metabolism are increasingly recognized as critical metabolic regulators, their interaction in osteosarcoma remains poorly understood. The autophagy-related protein GABARAP, central to mitochondrial quality control, has not been systematically evaluated in osteosarcoma.

**Methods:**

Single-cell RNA sequencing (scRNA-seq) datasets (GSE162454, GSE237070) were analyzed to delineate cellular heterogeneity and malignant states, with prognostic clusters identified by Scissor and inferCNV. Tumor microenvironment (TME) composition and intercellular signaling were profiled using CellChat. Pathway enrichment and multi-omics integration across TARGET, GSE21257, and GSE32981 highlighted mitophagy-pyruvate coupling, which were further validated by spatial transcriptomics and *in vitro* functional assays.

**Results:**

We mapped the osteosarcoma ecosystem and identified two malignant subpopulations, Ost_1 and Cho_2 (Mal_Ost/Cho), exhibiting high genomic instability, stemness, and poor prognosis. The osteosarcoma TME displayed profound immune remodeling, characterized by infiltration of T/NK cells alongside enrichment of immunosuppressive Tregs and M2-polarized macrophages. Enhanced MIF-mediated signaling between Mal_Ost/Cho and T/NK compartments suggested a key mechanism of immune evasion. Both malignant subtypes demonstrated coordinated activation of mitophagy and pyruvate metabolism, sustaining metabolic adaptation and tumor progression. Multi-omics integration pinpointed GABARAP as a central hub regulating this mitophagy-metabolism axis, spatially enriched within metabolic hotspots and immunosuppressive niches. Functionally, GABARAP depletion disrupted mitophagy flux, mitochondrial integrity, and energy production, thereby impairing osteosarcoma cell proliferation and migration.

**Conclusion:**

These findings reveal that GABARAP links mitophagy-driven metabolic adaptation with immune evasion, representing a key regulator and potential therapeutic target in osteosarcoma.

## 1 Introduction

Osteosarcoma represents the most prevalent primary malignant bone neoplasm, arising from mesenchymal progenitor cells with predilection for metaphyseal regions of long bones (1, 2). The disease demonstrates a distinct bimodal age distribution, with peak incidence observed in adolescents and young adults aged 10–20 years, followed by a secondary smaller peak in elderly patients around 60 years (3). Current standard-of-care regimens encompass neoadjuvant chemotherapy, surgical resection, and adjuvant chemotherapy, which have enhanced survival outcomes in localized disease (5-year survival: 60-70%) (4, 5). However, patients with recurrent or metastatic disease face dismal prognoses, with 5-year survival rates dropping to approximately 20% (6). These limitations underscore the critical need for novel therapeutic targets and strategies. Recent technological advancements in scRNA-seq and spatial transcriptomics have revolutionized our capacity to dissect tumor heterogeneity, revealing previously unrecognized molecular subtypes and spatially resolved interactions that demand more precise mechanistic insights for therapeutic innovation.

Increasing evidence highlights that metabolic reprogramming is a hallmark of osteosarcoma, where mitochondrial quality control and pyruvate metabolism cooperate to sustain malignant traits (7, 8). Mitophagy, a selective form of autophagy, eliminates dysfunctional mitochondria and preserves cellular homeostasis under stress. In osteosarcoma, pathways such as FOXO3a/HSP90/ULK1-FUNDC1 and the FoxG1/BNIP3 axis enhance mitophagy, protecting tumor cells from cisplatin-induced apoptosis and contributing to chemoresistance (9–11). Concurrently, pyruvate metabolism is rewired through upregulation of pyruvate dehydrogenase kinase-1 (PDK1) and lactate dehydrogenase-A (LDHA), which divert pyruvate toward glycolysis and lactate production, thereby promoting stemness, metastasis, and poor prognosis (12, 13). Monocarboxylate transporter-1 (MCT1) further modulates pyruvate-lactate shuttling, fueling invasive phenotypes (14). Integrative transcriptomic analyses have identified mitophagy-related genes as prognostic indicators in osteosarcoma, underscoring the tight coupling of mitochondrial turnover and pyruvate flux (15). Together, these findings suggest that mitophagy and pyruvate metabolism form a reciprocal regulatory network that supports osteosarcoma progression, therapy resistance, and metabolic adaptation, positioning this axis as a promising therapeutic target.

GABA type A receptor-associated protein (GABARAP), a ubiquitin-like member of the ATG8 family, was initially identified through its interaction with the γ2 subunit of the GABA_A_ receptor and was recognized as a key mediator of GABA_A_ receptor membrane trafficking and synaptic localization (16). Beyond its neural functions, GABARAP critically participates in intracellular trafficking, endocytic processes, and mitochondrial quality control (17). Through interactions with key signaling nodes including ULK1 kinase and the PI3K/AKT/mTOR pathway, GABARAP modulates cellular metabolic plasticity, stress adaptation, and survival mechanisms. As a core component of the autophagy machinery, GABARAP facilitates autophagosome maturation and lysosomal fusion, positioning it as a central regulator of mitophagy with dual oncogenic implications: while promoting tumor suppression in certain contexts, its overexpression has been linked to radio-resistance in hepatocellular carcinoma and enhanced carcinogen-induced tumorigenesis (18, 19). However, its precise functional dynamics and therapeutic relevance in osteosarcoma remain understudied, representing a critical gap in understanding mitophagy-driven oncogenic adaptations in this aggressive bone malignancy.

In this study, we identified two malignant subpopulations, Ost_1 and Cho_2, characterized by high stemness, genomic instability, and poor prognosis. The osteosarcoma microenvironment exhibited marked immune remodeling with T/NK infiltration, enrichment of Tregs and M2 macrophages, and enhanced MIF-mediated crosstalk driving immune evasion. Multi-omics analyses revealed coordinated activation of mitophagy and pyruvate metabolism as a core metabolic program, with GABARAP emerging as a central regulator sustaining mitophagy flux, pyruvate utilization, and malignant phenotypes. Its spatial enrichment in metabolic and immunosuppressive niches underscores its pathogenic significance, positioning GABARAP as a potential therapeutic target and biomarker of aggressive osteosarcoma.

## 2 Materials and methods

### 2.1 Data acquisition and preprocessing

This study integrated multi-omics datasets for osteosarcoma, including scRNA-seq, spatial transcriptomics, and bulk RNA-seq data. Firstly, scRNA-seq data originated from two published studies: GSE162454 (17) (DOI: 10.3389/fonc.2021.709210; sequenced with a 10× genomics plate) comprising six treatment-naïve primary tumor samples, and GSE237070 (18) (DOI: 10.1186/s12916-024-03319-w; sequenced with a 10× genomics plate) containing two primary tumors and two matched adjacent non-tumor tissues. Moreover, we also enrolled another integrating publicly available osteosarcoma scRNA-seq data (https://github.com/zhengxj1) with chemotherapy response information (20), which contained four chemotherapy-resistant and one chemotherapy-sensitive tumor samples. In addition, one spatial transcriptomic data was also obtained from the same source. The corresponding clinical information and sequencing platforms corresponding to the samples as above were summarized in **Supplementary Table S1**. Thirdly, the bulk RNA-seq datasets were retrieved from the TARGET (https://ocg.cancer.gov/programs/target) as well as GEO datasets (GSE21257 and GSE32981).

### 2.2 scRNA-seq data processing and cell type annotation

The osteosarcoma scRNA-seq data were systematically processed using the Seurat (v4.1.1) R package. First, a Seurat object was created using the CreateSeuratObject function, with the min. cells parameter set to 3 to exclude genes expressed in fewer than three cells. Subsequently, further filtering of the cell data was performed, which involved removing cells with fewer than 200 or more than 8000 detected genes, as well as cells exhibiting a mitochondrial gene proportion exceeding 10% or a hemoglobin gene proportion exceeding 5%. To minimize the impact of doublets, the doubletFinder v3 function from the DoubletFinder package was used to identify and filter potential doublets. Key parameters were set as PCs = 1:20 and pN = 0.25, meaning that 20 principal components were considered to estimate the probability of each cell being classified as a doublet at 0.25. The filtered data were subsequently normalized using the LogNormalize method, wherein the raw counts were scaled to a total gene expression of 10,000 per cell. Subsequently, 2,000 highly variable genes were identified using the FindVariableFeatures function, followed by normalization with the ScaleData function to mitigate the impact of technical noise. Dimensionality reduction was then performed using the RunPCA function, with the first 20 principal components selected for subsequent analyses. For batch effect correction in multi-sample data, the RunHarmony function of the Harmony package was used for data integration. The samples were treated as the grouping variable (group.by.vars = “sample”), with the integration strength parameter set to lambda = 1 and the clustering penalty parameter set to theta = 2. The Harmony method, based on Principal Component Analysis (PCA), corrects for batch effects by embedding and iteratively removing systematic biases specific to each dataset, enabling effective integration of cells from different samples so that they cluster together. Subsequently, Uniform Manifold Approximation and Projection (UMAP) dimensionality reduction was performed using the ‘umap-learn’ algorithm in the RunUMAP function to facilitate subsequent visualization of the integrated data. After batch effect correction, the FindNeighbors function was used to calculate the distances between cells and construct a Shared Nearest Neighbor (SNN) graph. Cell clustering was then performed using the FindClusters function using the Louvain algorithm with a resolution parameter set to 0.3 to identify cell subpopulations. Finally, during the cell annotation phase, the automated annotations generated by the SingleR software were combined with known cell marker genes (**Supplementary Table S2**) and manual corrections were made to further refine the annotations.

### 2.3 Functional enrichment analysis

Differentially expressed genes (DEGs) were identified for each cluster using Seurat’s FindMarkers function (v4.0.0), focusing on upregulated genes with an FDR < 0.05 to ensure statistical significance. Functional enrichment analyses, including Gene Ontology (GO) and Kyoto Encyclopedia of Genes and Genomes (KEGG) pathway analysis, were performed using the clusterProfiler R package (v4.8.2) (21). GO enrichment analysis categorized DEGs into biological processes, molecular functions, and cellular components using the enrichGO function, while KEGG pathway analysis identified associated metabolic and signaling pathways with the enrichKEGG function. Significant results were filtered at an FDR < 0.05. To visualize the enriched terms and pathways, bar plots were generated using the barplot functions from clusterProfiler. Additionally, gene-set enrichment analysis (GSEA) was conducted to explore dysregulated molecular pathways, utilizing pre-defined gene sets for a broader analysis. All statistical analyses were performed in R (v4.1.1), and visualizations were created using ggplot2.

### 2.4 Identification of malignant versus non-malignant osteoblasic/chondroblastic cells

To distinguish malignant from non-malignant osteoblastic and chondroblastic cells, we employed the infercnvpy Python package, which identifies copy number variations (CNVs) based on large-scale genomic data. CNVs were assessed across cells within the TME to detect chromosomal aberrations distinguishing malignant cells from non-malignant populations. Cells from the TME, specifically T and NK cells, were used as normal reference populations, providing a baseline for expected CNV patterns in non-malignant cells. The CNV score for each cell was calculated, with cells exhibiting a CNV score greater than 0.01 classified as malignant and those with a score less than 0.01 identified as non-malignant. This threshold enabled a clear distinction between malignant and normal osteoblasts and chondroblasts. The results were visualized through CNV boxplots, which allowed us to further validate the classification and confirm the presence of genomic aberrations characteristic of malignant cells within the TME.

### 2.5 Identification of prognostically relevant cellular subtypes

We implemented the Scissor algorithm (v2.0.0) to integrate scRNA-seq data with clinical survival annotations from the TARGET-OS bulk RNA-seq cohort (22), applying parameters α = 0.01 and family = ‘binomial’ to osteogenic and chondrogenic cell subsets. Cells exhibiting positive correlation with adverse survival outcomes were designated as “worse survival” subpopulations, while those demonstrating favorable prognostic associations were classified as “good survival” clusters through Cox proportional hazards modeling.

### 2.6 Pseudotime trajectory inference

The Monocle 2 package (v2.28.0) was used to construct pseudotime trajectories of osteogenic and chondrogenic cells based on their transcriptional profiles (23). After dimensionality reduction and cell ordering, cells were projected onto developmental trajectories with branching structures, where cells within the same branch were inferred to share a similar cellular state. Branched expression analysis modeling (BEAM) was further applied to identify branch-specific genes that may play critical roles in cell fate decisions.

### 2.7 Cell-cell communication analysis

We investigated intercellular communication using the CellChat R package (v1.6.1) (24). A merged Seurat object containing osteoblasts, chondroblasts and TME-derived cells was used as input. A curated signaling network was constructed based on known ligand-receptor interactions. Intercellular communication probability and pathway-level interactions were inferred using the computeCommunProb and computeCommunProbPathway functions.

### 2.8 Spatial transcriptomic integration and spatial deconvolution

The spatial transcriptomic data was obtained from the GitHub repository (19), including 1 osteosarcoma samples with chemotherapy resistance at diagnosis. For this spatial transcriptomics data, spots with less than 200 genes or mitochondrial transcripts greater than 20% were discarded, and genes expressed in less than 2 spots were removed. Count matrix was standardized with SCTransform function in Seurat package (v4.1.1) to account for variance in sequencing depth across data points and detect high-variance features. Based on the pathology annotation for tissue regions, we identified region-specific marker genes through FindAllMarkers function (logFC.threshold > 1 and adjusted p-values < 0.05). To investigate the spatial location of malignant osteoblastic and chondroblastic cells in osteosarcoma, we employed CARD (25) (version 1.1) method to deconvolute the spatial transcriptomics data based on the single-cell data of malignant osteoblastic and chondroblastic cells. The CARD performed deconvolution through a non-negative factorization framework and output the estimated composition of malignant osteoblastic and chondroblastic cells across spatial locations with two inputs, which included the single-cell transcriptional profiles of all CCSs and the ST data with localization information. The CARD_deconvolution function with default parameters was utilized to calculate the proportion of cells containing malignant osteoblastic and chondroblastic cells at each spatial location.

### 2.9 Cell culture, transfection and viral infection

Human osteosarcoma cell lines 143B (ATCC, CRL-8303) and U2OS (ATCC, HTB-96), as well as the human embryonic kidney cell line 293T (ATCC, CRL-1573), were obtained from the American Type Culture Collection (ATCC). To generate stable GABARAP-silenced osteosarcoma cells, lentiviral shRNA plasmids targeting GABARAP in the plKO.1 vector were constructed by Tsingke Biotechnology (Nanjing, China). The lentiviral supernatants were collected from 293T cells transfected with second-generation virus packaging system psPAX2 (Addgene Inc. 12260, Watertown, MA, USA) and PMD2.G (Addgene Inc. 12259, Watertown, MA, USA) plasmids, and infected cells using 10 mg/mL polybrene (MedChemExpress, HY-112735, South Brunswick, NJ, USA) for 48 h. Then the stably transfected cells were selected with 2 μg/ml puromycin (Thermo Fisher Scientific, A1113803, Inc. Waltham, MA, USA) for 7 days. Gene silencing efficiency was verified by real-time quantitative PCR (RT-qPCR) assays. The PCR Primers and shRNA sequences were listed in **Supplementary Table S3**. All cells were cultured in Dulbecco’s modified Eagle medium (DMEM; Cellmax, CGM101.05, Beijing, China) supplemented with 10% heat-inactivated fetal bovine serum (FBS; Cellmax, SA211.02, Beijing, China) and 1% penicillin/streptomycin (P/S, Gibco, 15140-122, Waltham, MA, USA) under standard conditions: a humidified incubator with 5% CO_2_ at 37°C.

### 2.10 Cell viability assay

The viability of osteosarcoma cells was determined by Cell Counting Kit 8 (CCK-8, 40203ES60, Yeasen, Shanghai, China) assay. In brief, 2× 10^3^ osteosarcoma cells were seeded into 96-well plates and then cultured at 37°C in a humidified atmosphere with 5% CO2. After culturing for 0, 24, 48, 72 and 96 h, the optical density of each well was measured with CCK-8 kit. The absorbance was measured at 450 nm using a microplate reader (BioTek Instruments, USA).

### 2.11 Colony formation assays

For the colony formation assay, osteosarcoma cells were seeded into 6-well plates with 500 cells per well. After two weeks, the cells were fixed in 4% paraformaldehyde for 20 minutes and stained with 0.1% crystal violet for another 20 minutes. Colony images were captured using an optical microscope (Olympus, Tokyo, Japan), and colony numbers were quantified using ImageJ software.

### 2.12 Wound healing assay

Osteosarcoma cells were cultured to full confluence in 6-well plates. Then, cells were gently scratched with a 20-μl micropipette tip in the center of the well, followed by incubating with serum-free medium. Images were captured at 0 and 24 hours using an optical microscope (Olympus, Tokyo, Japan). The width of wound healing was quantified and compared with baseline values.

### 2.13 Mitophagy detection by immunofluorescence

Osteosarcoma cells were prepared in confocal Petri dishes. Mitochondria of live cells were stained with 200 nM MitoTracker Red CMXRos (Beyotime, 40743ES50, Shanghai, China) working solution for 20 minutes at 37°C. The cells were then further stained with 100 nM LysoTracker Green (Beyotime, C1047S, Shanghai, China) working solution for 60 minutes at 37°C. After three washes with PBS, cells were counterstained with Hoechst33342. Fluorescence images were captured using a confocal laser scanning microscope (LSM 800; Zeiss, Germany).

### 2.14 Pyruvate and ATP measurement

The intracellular pyruvate and ATP levels were quantified using the Amplex Red Pyruvate Assay Kit (Beyotime, S0299S, Shanghai, China) and the ATP Assay Kit (Beyotime, S0026, Shanghai, China), respectively. Cells were seeded in 6-well plates and cultured for 24 hours, followed by centrifugation at 12,000 × g for 5 minutes to collect the supernatants. The OD values was measured at 570 nm (pyruvate) and 560 nm (ATP) using a microplate reader (BioTek Instruments, USA).

### 2.15 Statistical analysis

Statistical analyses were performed using GraphPad Prism 8.0.2 (GraphPad Software, La Jolla, CA, USA). Differences between groups were evaluated using unpaired Student’s t-test or one-way ANOVA. For non-parametric comparisons, the Mann-Whitney U test (two groups) or Kruskal-Wallis test (multiple groups) was applied. Categorical data were analyzed using the Chi-square test. Survival differences were assessed using Kaplan-Meier curves and the log-rank test. All data are presented as mean ± standard deviation (SD) from at least three independent biological replicates. Statistical significance was defined as P < 0.05 unless otherwise specified.

## 3 Results

### 3.1 Construction of single cell atlas of osteosarcoma

**Figure 1:**
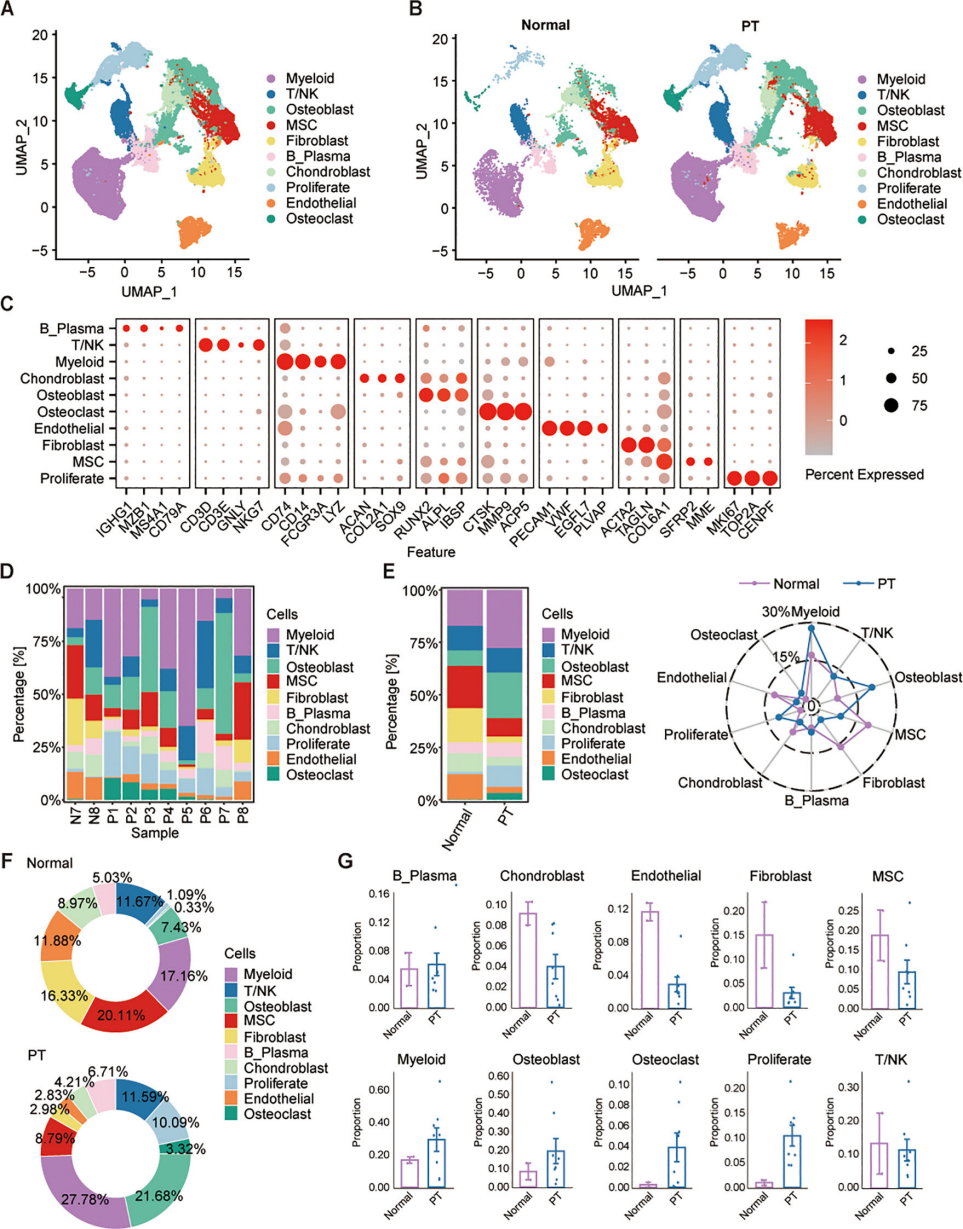
Single-cell transcriptome profiles of osteosarcoma tumor and adjacent non-tumor samples. **(A, B)** UMAP projections identify 10 major cell populations across osteosarcoma and adjacent normal tissues. **(C)** Dot plot shows the expression of canonical marker gene across different cell types. **(D, E)** Cell-type proportions vary across patients and between normal and tumor tissues. **(F)** Pie plots show the relative proportions of each cell type in the normal (top) and PT (bottom) groups, with percentages indicated for each cell type. **(G)** Box plots show the percentage of each cell type in normal and PT, respectively.

To investigate the cellular architecture and molecular heterogeneity of osteosarcoma, we performed scRNA-seq analysis on 10 patient-derived samples retrieved from the GEO database (accession numbers GSE162454 and GSE237070). This cohort comprised eight osteosarcoma primary tumor (PT) specimens and two matched adjacent normal tissues (**Figure 1A**). Following rigorous quality control and doublet removal, we obtained 83,228 high-quality single-cell transcriptomes, with 62,417 cells derived from PT tissues and 20,811 cells from normal samples (**Figure 1B**). Unsupervised graph-based clustering combined with UMAP visualization identified ten distinct cellular populations, which were annotated using established marker genes: osteoblasts (RUNX2, ALPL, IBSP), chondroblasts (ACAN, COL2A1, SOX9), myeloid cells (CD74, CD14), T/NK cells (CD3D, CD3E), mesenchymal stem cells (MSCs) (COL6A1), fibroblasts (ACTA2, TAGLN), B cells (IGHG1, MZB1), proliferative cells (MKI67, TOP2A, CENPF), endothelial cells (PECAM1), and osteoclasts (CTSK) (**Figure 1C**). Inter-sample comparison revealed high heterogeneity in cellular composition across individual patients (**Figure 1D**). Cell type proportions differed notably among the two groups including myeloid (27.78%), osteoblast (21.68%), osteoclast (3.32%) and proliferate cells (10.09%) were enriched in PT group, while chondroblasts (8.97%), endothelial (11.88%), fibroblasts (16.33%) and MSCs (20.11%) were markedly increased in normal tissues (**Figures 1E-G**). These findings highlight the dynamic cellular reorganization occurring during osteosarcoma progression, with specific cell types undergoing expansion or depletion in the tumor microenvironment.

### 3.2 Characterization of the landscapes for T/NK Cells and fibroblasts across osteosarcoma

**Figure 2:**
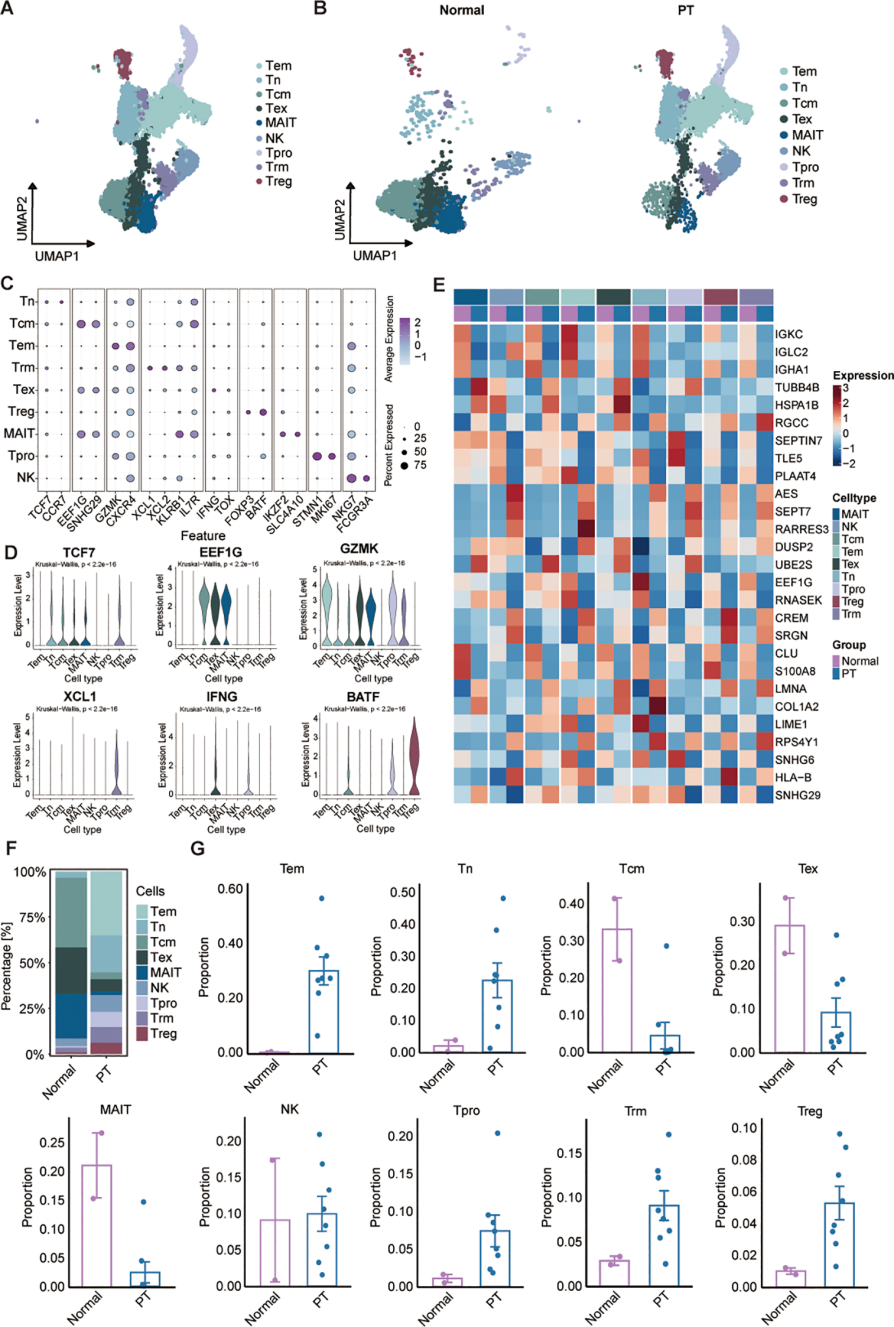
Characterization of the landscapes for T/NK cells. **(A)** UMAP of T/NK cells showing major subtypes. Tem: the effector memory T cells; Tn: the naïve T cells; Tcm: the central memory T cells; Tex: the exhausted T cells; MAIT: the mucosal-associated invariant T cells; NK cells; Tpro: the proliferating T cells; Trm: the tissue-resident memory T cells; Treg: the regulatory T cells. **(B)** UMAP plot showing T/NK cell subsets of normal (left) and PT (right) groups. **(C)** Dot plot of selected marker gene expression across T/NK subsets. **(D)** Violin plots showing the normalized expression levels of six representative marker genes across the nine T/NK subsets. **(E)** Heatmap of DEGs across T/NK clusters between normal and PT samples. **(F)** T/NK subtype frequencies across normal and PT groups. **(G)** Box plots show the percentage of nine T/NK cell types in normal and PT, respectively.

Given the central role of T/NK cells in tumor immunity, we initially characterized this compartment using unsupervised clustering, identifying nine major subsets annotated via canonical markers (**Figure 2A**). These clusters were resolved within both PT and normal tissue groups (**Figure 2B**). Expression profiles of representative lineage-specific genes across the populations are shown (**Figures 2C, D**): (1) the effector memory T cells (Tem) highly expressing GZMK, and CXCR4; (2) the naïve T cells (Tn) highly expressing TCF7, and CCR7; (3) the central memory T cells (Tcm) characterized with high EEF1G, and SNHG29 expression; (4) the exhausted T cells (Tex) specifically express the markers IFNG and TOX; (5) the mucosal-associated invariant T cells (MAIT) with high expression of IKZF2 and SLC4A10; (6) the NK cells specifically expressing NKG7 and FCGR3A; (7) the proliferating T cells (Tpro) highly expressing cell proliferating marker and T cell markers MKI67 and STMN1; (8) the tissue-resident memory T cells (Trm) highly expressing XCL1, XCL2, and KLRB1; (9) the regulatory T cells (Treg) expressing FOXP3, and BATF (**Figures 2C, D**). Additional discriminatory markers between normal and tumor tissues were in **Figure 2E**; notably, Tregs in PT exhibited elevated expression of immunosuppression-associated genes AES, RARRES3, SRGN, and CREM within the osteosarcoma TME. Analysis of cellular composition revealed normal tissues dominated by MAIT (24.40%), Tcm (38.1%), and Tex (25.38%), with low frequencies of Tem (0.68%), Tn (2.90%), Tpro (0.77%), Treg (0.89%), and Trm (2.60%). Tumors exhibited enrichment of Tem (35.06%), Tn (20.34%), and NK (9.16%) cells, with Treg proportion (6.17%) remaining elevated versus normal (**Figures 2F, G**). These results further demonstrate immune infiltration within osteosarcoma tissues, coupled with a pronounced enrichment of immunosuppressive Tregs.

We next analyzed fibroblasts, a major stromal component in osteosarcoma exhibiting significant heterogeneity. Unsupervised subtyping resolved five distinct fibroblast populations defined by unique marker gene signatures: inflammatory cancer-associated fibroblasts (iCAFs: high APOD, IGF1, ITM2A), matrix-producing cancer-associated fibroblasts (mCAFs: high MMP11, COL1A1, POSTN), antigen-presenting cancer-associated fibroblasts (apCAFs: high CD74, HLA-DPB1, HLA-DRA), pericytes (HIGD1B, FRZB, MT1A), and smooth muscle cells (SMCs: high CLSTN2, ADGRL3) (**Supplementary Figures S1A-D**). **Supplementary Figure S1E** displayed the top five differentially expressed genes per cell type between normal and tumor tissues. As depicted, the heatmap revealed that mCAFs in the tumor group showed markedly elevated expression of POSTN, PRSS23 and PLXDC1, which were associated with tumor infiltration and immune suppression (**Supplementary Figure S1E**). Cellular abundance analysis demonstrated pronounced enrichment of mCAFs (68.35%) in PT tissues versus normal, implicating their role in extracellular matrix remodeling and progression (**Supplementary Figures S1F, G****).** Conversely, iCAFs (1.85%), characterized by inflammatory mediator expression, were significantly reduced in tumors, potentially reflecting suppressed inflammatory signaling impacting immune cell recruitment (**Supplementary Figures S1F, G**). Collectively, these altered fibroblast subpopulation profiles suggest contributions to osteosarcoma TME progression and immunosuppressive niche formation.

### 3.3 Osteosarcoma exhibits a tumor−associated macrophage subpopulation

**Figure 3:**
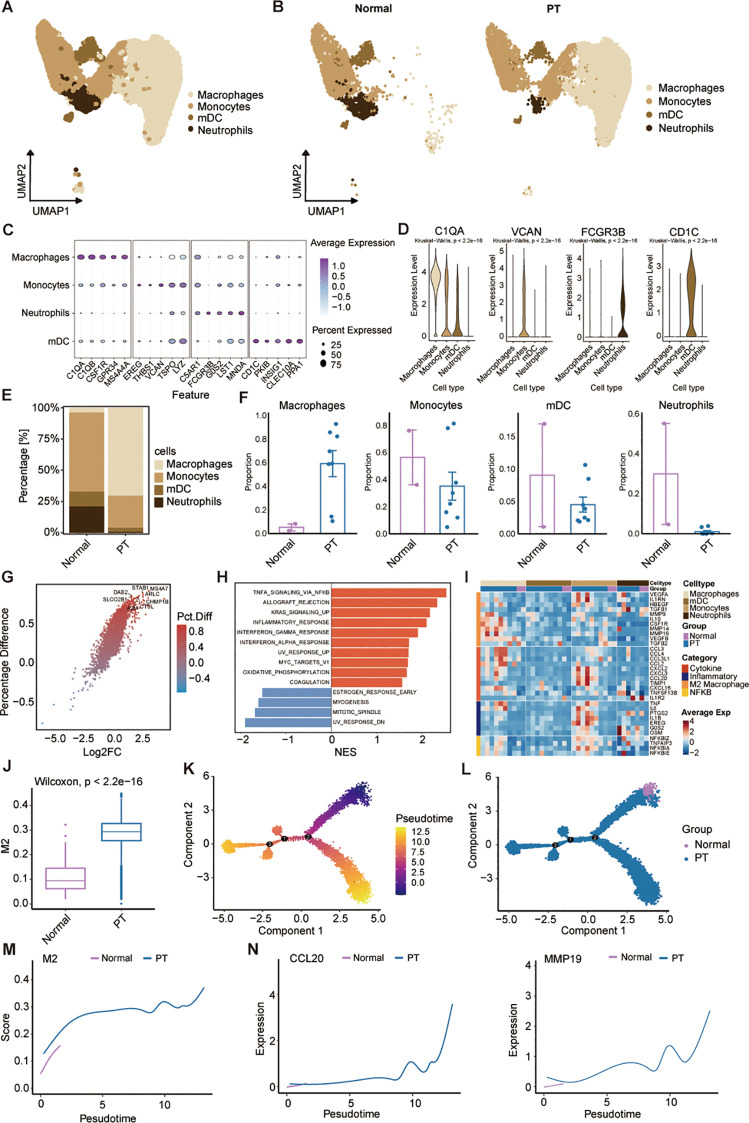
Characterization of the landscapes for myeloid cells. **(A)** UMAP of myeloid cells. Monocytes; macrophages; mDCs: myeloid dendritic cells; and neutrophils. **(B)** UMAP visualization of myeloid cell populations in normal and primary tumor tissues. **(C)** Dot plot showing expression of representative marker genes across myeloid cell subsets; dot size indicates percentage of expressing cells, and color indicates average expression level. **(D)** Violin plots showing the normalized expression levels of six representative marker genes across the four myeloid cell subsets. **(E)** Myeloid cell subtype frequencies across normal and PT groups. **(F)** Box plots show the percentage of four myeloid cell types in normal and PT, respectively. **(G)** Volcano plot comparing differentially expressed genes of macrophage between normal and PT groups. **(H)** The bar plot of HALLMARK enrichment differences between normal group and PT groups. **(I)** Heatmap shows average expression of select genes from different categories (rows) across different cell populations (top color bar) for each patient (columns). **(J)** Box plot showing M2 scores of macrophages in the normal and PT groups. **(K)** Pseudotime plot showing the trajectory of all macrophages between normal and PT groups. **(L)** Trajectory plot indicating the locations of macrophages with normal and primary tumor groups. **(M, N)** Trajectory plot indicating the locations of M2 score and specific genes (CCL20 and MMP19) with normal and primary tumor groups.

Myeloid cells constitute a critical and abundant heterogeneous population within the osteosarcoma TME, encompassing subtypes with both tumor-promoting and suppressive functions. Unbiased clustering of the cells identified four distinct myeloid subclusters, including macrophages, monocytes, myeloid dendritic cells (mDCs), and neutrophils according to their gene profiles and canonical markers (**Figures 3A, B**). Expression profiling of signature genes revealed specific characteristics for each subcluster (**Figures 3C, D**): (1) Macrophages highly expressing C1QA, CSF1R, and MS4A4A; (2) Monocytes displayed relatively high VCAN and TSPO expression; (3) Neutrophils showed high MNDA levels; (4) mDCs were characterized by elevated expression of CD1C, INSIG1, and CLEC10A. Comparative analysis of cell abundance revealed a pronounced shift in tumor tissues, where macrophages constituted the dominant population (70.36%), while monocytes (25.68%), mDCs (3.27%), and neutrophils (0.69%) were significantly depleted relative to normal tissue (**Figures 3E, F**). This shift indicates enhanced monocyte-to-macrophage differentiation within the TME, potentially contributing to immunosuppression and osteosarcoma progression. To assess the transcriptomic changes in macrophages, we performed pairwise DEGs analysis. There were 1677 differentially-expressed genes (DEGs) (|Log fold-change| > 0.25, adjusted p-value < 0.05). We further filtered the DEGs by comparing the percent of normal and PT macrophage cells that express each gene (calculating the percentage difference) (**Figure 3G**). A summary of the top eight upregulated DEGs within PT macrophage cells was shown (**Figure 3G**). Gene Set Enrichment Analysis (GSEA) further demonstrated significant enrichment in the tumor group for pathways including TNFA_SIGNALING_VIA_NFKB, KRAS_SIGNALING, and INFLAMMATORY_RESPONSE compared to the normal group (**Figure 3H**), indicating a pro-tumor role for macrophages in osteosarcoma progression.

Additionally, we further analyzed macrophage-related gene expression signatures, including cytotoxicity, inflammation, M2 score, and NF-κB across samples (**Figure 3I**). Macrophages in PT tissues exhibited a transcriptional profile characteristic of M2 polarization (**Figure 3I**), a phenotype known to suppress anti-tumor immunity across diverse malignancies (26). Consistent with this phenotype, PT-associated macrophages expressed inflammatory cytokines (TNF, IL6) and pro-tumorigenic factors (CCL2, CCL3, CCL4, CXCL16) linked to cancer progression and invasiveness (**Figure 3I**). To specifically assess the contribution of M2 polarization, we compared macrophage M2 scores between PT and normal tissues. PT samples displayed significantly elevated M2 scores (**Figure 3J**). Pseudotemporal trajectory analysis using Monocle2 revealed a positive correlation between increasing M2 scores and macrophage differentiation from normal to PT states (**Figures 3K-M**). Concomitantly, expression of established M2 markers CCL20 and MMP9 increased during this differentiation (**Figure 3N**), confirming the predominant M2 phenotype of tumor-infiltrating macrophages. Collectively, these data demonstrate myeloid reprogramming within the osteosarcoma TME, dominated by M2-polarized macrophages that drive immunosuppression and tumor progression.

### 3.4 A mitophagy-driven osteoblastic cell state initiates osteosarcoma progression

**Figure 4:**
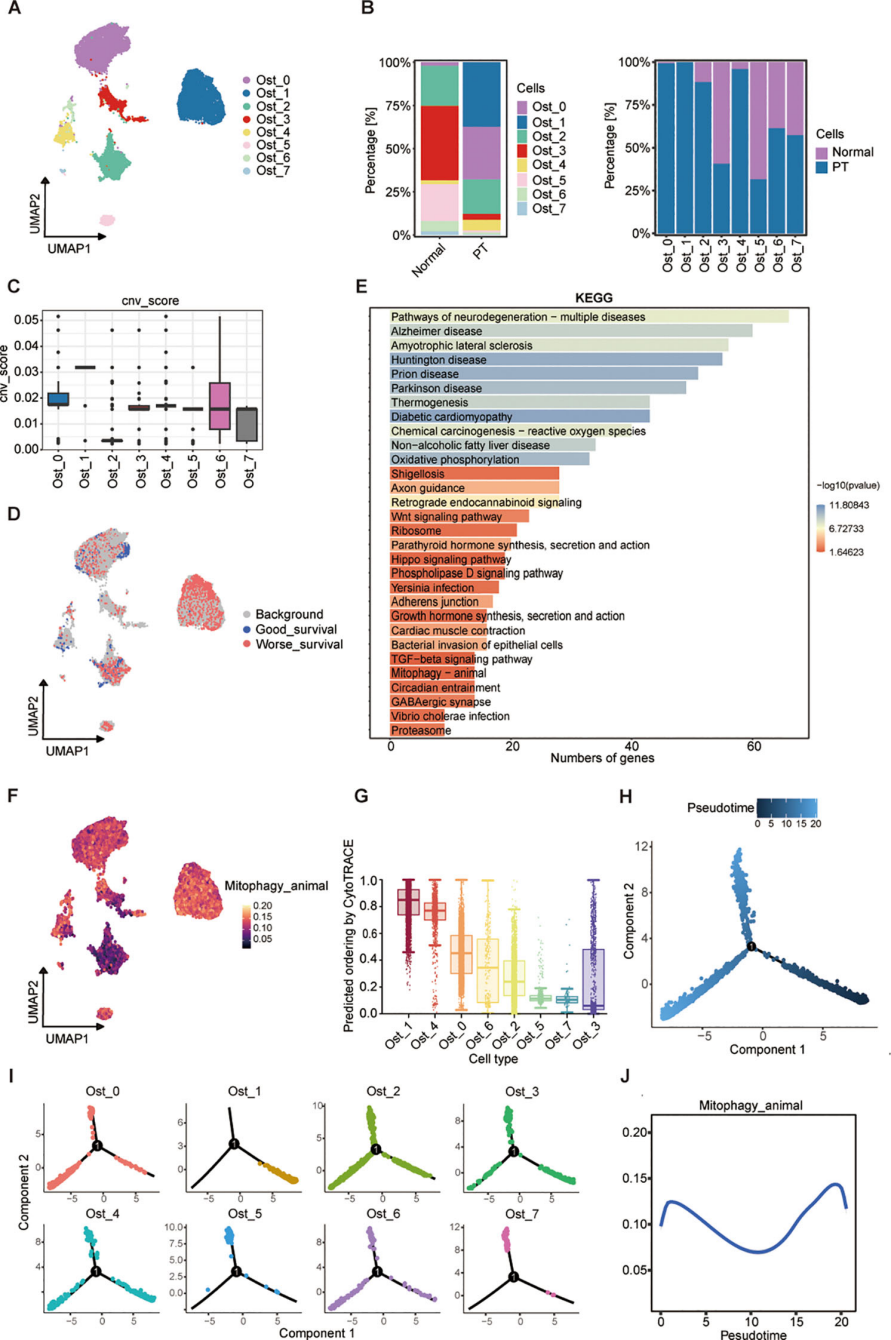
A mitophagy-driven osteoblastic cell state initiates osteosarcoma progression. **(A)** UMAP plot showing eight osteoblast clusters (Ost_0-Ost_7) identified by unsupervised clustering. **(B)** Relative proportions of each cluster in normal and PT tissues (left), and their distribution within individual conditions (right). **(C)** CNV scores inferred from transcriptomic data across clusters. **(D)** UMAP embedding colored by survival-associated gene expression signatures, stratified by good versus worse prognosis. **(E)** KEGG pathway enrichment analysis of differentially expressed genes across clusters; bar length represents number of genes, and color indicates -log10(P-value). **(F)** AUCell of mitophagy pathway activity across osteoblast clusters. **(G)** Predicted differentiation potential of each cluster inferred by CytoTRACE; higher values indicate greater stemness. **(H)** Pseudotime trajectory reconstruction of osteoblast populations using Monocle2, showing three major branches. **(I)** Pseudotime mapping of individual clusters onto the trajectory. **(J)** Dynamic changes in mitophagy pathway activity along pseudotime.

To elucidate the cellular heterogeneity of osteoblast-lineage cells in osteosarcoma, we performed UMAP analysis (**Supplementary Figure S2A**), and identified eight transcriptionally distinct osteoblastic subpopulations (Ost_0 to Ost_7) across primary tumor (PT) and normal cohorts (**Figure 4A**; **Supplementary Figure S2B**). Notably, Ost_0 and Ost_1 exhibited near-exclusive detection in tumor specimens (**Figure 4B**), implying tumor-specific identities. InferCNV-based copy number variation (CNV) profiling revealed Ost_1 possessed the highest CNV burden, indicative of pronounced genomic instability (**Figure 4C**; **Supplementary Figure S2C**). Based on the CNV score, we defined the osteoblastic subpopulations to malignant or not, and found that Ost_0 and Ost_1 as predominantly malignant, contrasting sharply with the minimal malignancy observed in Ost_2 (**Supplementary Figure S2D**). Integration of scRNA-seq data with clinical outcomes *via* the Scissor algorithm further validated their malignant potential, from which we found that among the 1,187 osteoblast-like cells which were associated with poor prognosis, 750 (63.2%) belonged to the Ost_1 cluster (**Figure 4D**; **Supplementary Figure S2E****),** suggesting that Ost_1 cluster is the dominant cell in osteosarcoma progression. Therefore, we compared the Ost_1 cluster versus other osteoblastic subpopulations through KEGG enrichment analyses, which highlighted mitophagy as a key pathway upregulated in Ost_1 (**Figure 4E**). Moreover, AUCell scoring confirmed elevated mitophagy activity in Ost_1 compared to other osteoblastic subsets (**Figure 4F**; **Supplementary Figure S2F**), underscoring its role in sustaining mitochondrial homeostasis and tumor cell survival. Additionally, CytoTRACE-derived stemness scores demonstrated that Ost_1 comprises early-stage, undifferentiated cells with tumor-initiating potential (**Figure 4G**; **Supplementary Figure S2G**). Monocle2-based trajectory inference consistently revealed a bifurcation point in osteoblast differentiation, with Ost_1 positioned at the root of the trajectory and Ost_3 representing a terminal differentiated state (**Figures 4H-I**; **Supplementary Figures S2H, I**).

Then, we adopted branched expression analysis modeling (BEAM) and identified 50 branch-specific genes clustered into six modules, with Module 4 enriched for mitochondrial transmembrane transport genes (**Supplementary Figure S2J**). Concurrent upregulation of genes related to mitochondrial inner membrane function, energy metabolism, and oxidative stress response suggested mitochondrial reprogramming as a driver of malignant transformation. Phosphatidylinositol-3,4,5-trisphosphate-binding gene enrichment implicated PI3K/AKT pathway activation, while MAPK and ECM-receptor interaction pathways were also upregulated, consistent with their roles in tumor proliferation and microenvironment remodeling (**Supplementary Figures S2K, L**). Dynamic profiling of mitophagy activity along the pseudotemporal trajectory revealed a biphasic pattern, with peaks at early and late stages and reduced activity during intermediate differentiation (**Figure 4J**). This suggests dual functional roles: maintaining mitochondrial integrity during early malignant transformation and mitigating oxidative damage to promote survival in advanced stages. Collectively, these findings identify a malignant osteoblastic subpopulation (Ost_1) as a key driver of osteosarcoma progression through transcriptional reprogramming, genomic instability, and mitochondrial adaptation.

### 3.5 A malignant chondroblast subpopulation driving osteosarcoma progression

**Figure 5:**
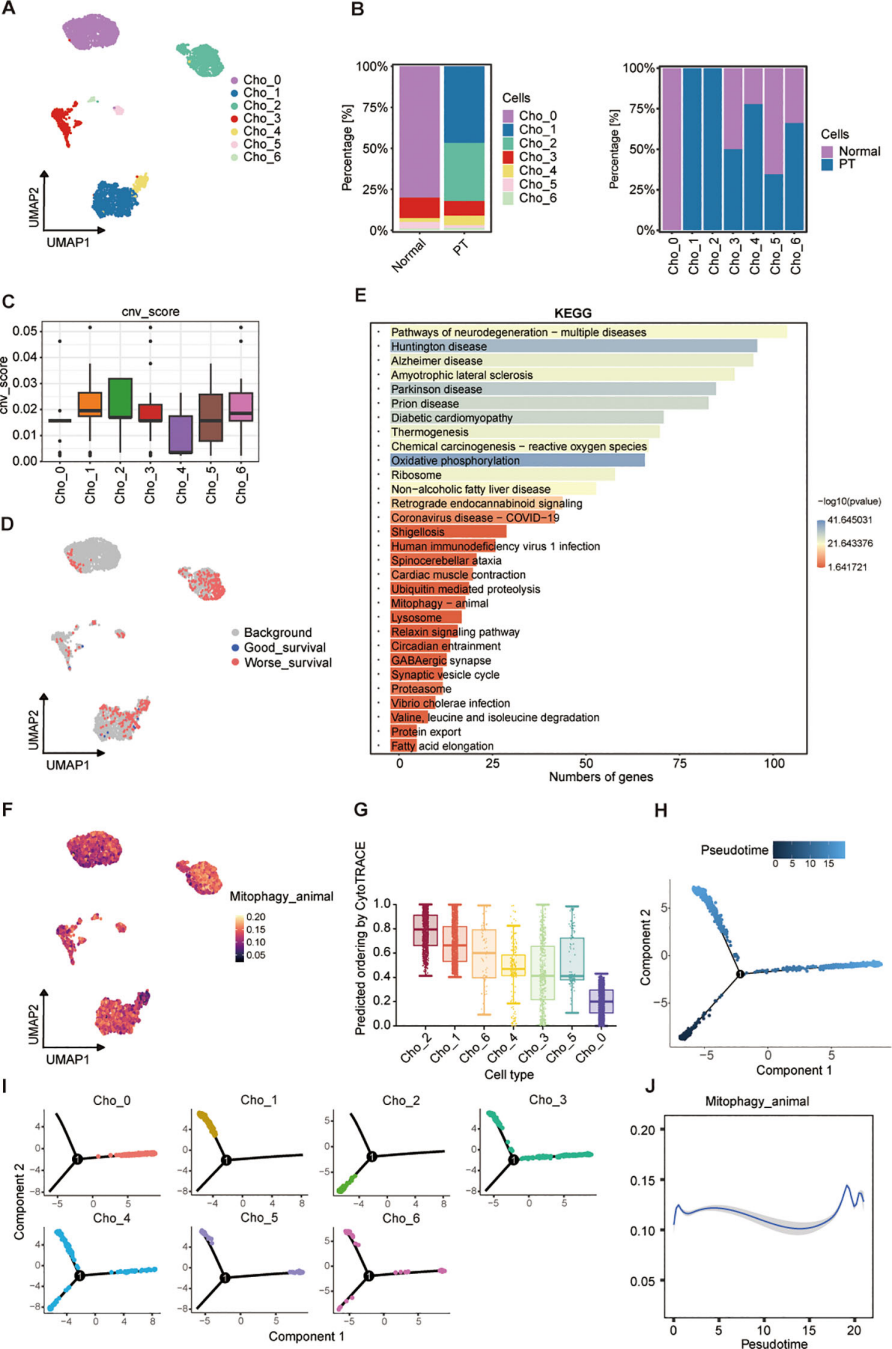
Transcriptional landscape and trajectory of chondroblast subpopulations. **(A)** UMAP plot of seven chondroblast clusters (Cho_0-Cho_6). **(B)** Proportional distribution of clusters in normal and PT tissues (left) and by condition (right). **(C)** CNV scores across clusters. **(D)** Cluster-level mapping of survival-related gene signatures. **(E)** KEGG pathway enrichment analysis of differentially expressed genes. **(F)** UMAP visualization of mitophagy pathway activity. **(G)** CytoTRACE-inferred differentiation potential of clusters. **(H)** Pseudotime trajectory of chondroblast populations. **(I)** Pseudotime projection of individual clusters. **(J)** Dynamics of mitophagy pathway activity along pseudotime.

Given evidence implicating chondroblasts in osteosarcoma progression through interactions with osteoblasts and osteogenic differentiation potential (27), we investigated chondroblasts as therapeutic targets. UMAP analysis identified seven transcriptionally heterogeneous chondroblast clusters (Cho_0-Cho_6) across PT and normal tissues (**Figure 5A**; **Supplementary Figures S3A, B**). Cho_1 and Cho_2 demonstrated pronounced tumor specificity, being virtually absent in normal tissue (**Figure 5B**). InferCNV analysis revealed substantially elevated genomic instability within these two clusters (**Figure 5C**; **Supplementary Figure S3C**). Malignancy classification based on CNV scores confirmed Cho_1 and Cho_2 as predominantly malignant, contrasting with the minimal malignancy observed in Cho_4 (**Supplementary Figure S3D**). Scissor algorithm-based integration of scRNA-seq data with clinical outcomes validated malignant potential: among 450 poor prognosis-associated chondroblast-like cells, 228 (50.7%) belonged to the Cho_2 cluster (**Figure 5D**; **Supplementary Figure S3E**), identifying Cho_2 as a dominant malignant chondroblast subset. KEGG enrichment analysis similarly highlighted mitophagy as a key pathway upregulated in Cho_2 (**Figure 5E**). Elevated mitophagy activity in Cho_2 relative to other chondroblast clusters was confirmed by AUCell scoring (**Figure 5F**; **Supplementary Figure S3F**). CytoTRACE analysis demonstrated Cho_2 comprises early-stage, undifferentiated cells possessing tumor-initiating potential (**Figure 5G**; **Supplementary Figure S3G**). Monocle2- derived time trajectory analysis revealed a consistent differentiation bifurcation, positioning Cho_2 at the trajectory root and Cho_0 at a terminally differentiated state (**Figures 5H, I**; **Supplementary Figures S3H, I**). BEAM analysis resolved six gene modules, encompassing pathways regulating bone development, osteoblast differentiation, and PI3K/Akt signaling (**Supplementary Figures S3J-L**). Notably, conserved mitophagy dynamics across both chondroblast and osteoblast lineages further implicated mitochondrial adaptation in malignant progression (**Figure 5J**). These findings establish malignant chondroblasts, particularly Cho_2, as actionable therapeutic targets in osteosarcoma *via* their inherent genomic instability, metabolic reprogramming, and differentiation plasticity.

### 3.6 The MIF-mediated intercellular communication axis enables immune evasion in osteosarcoma

**Figure 6:**
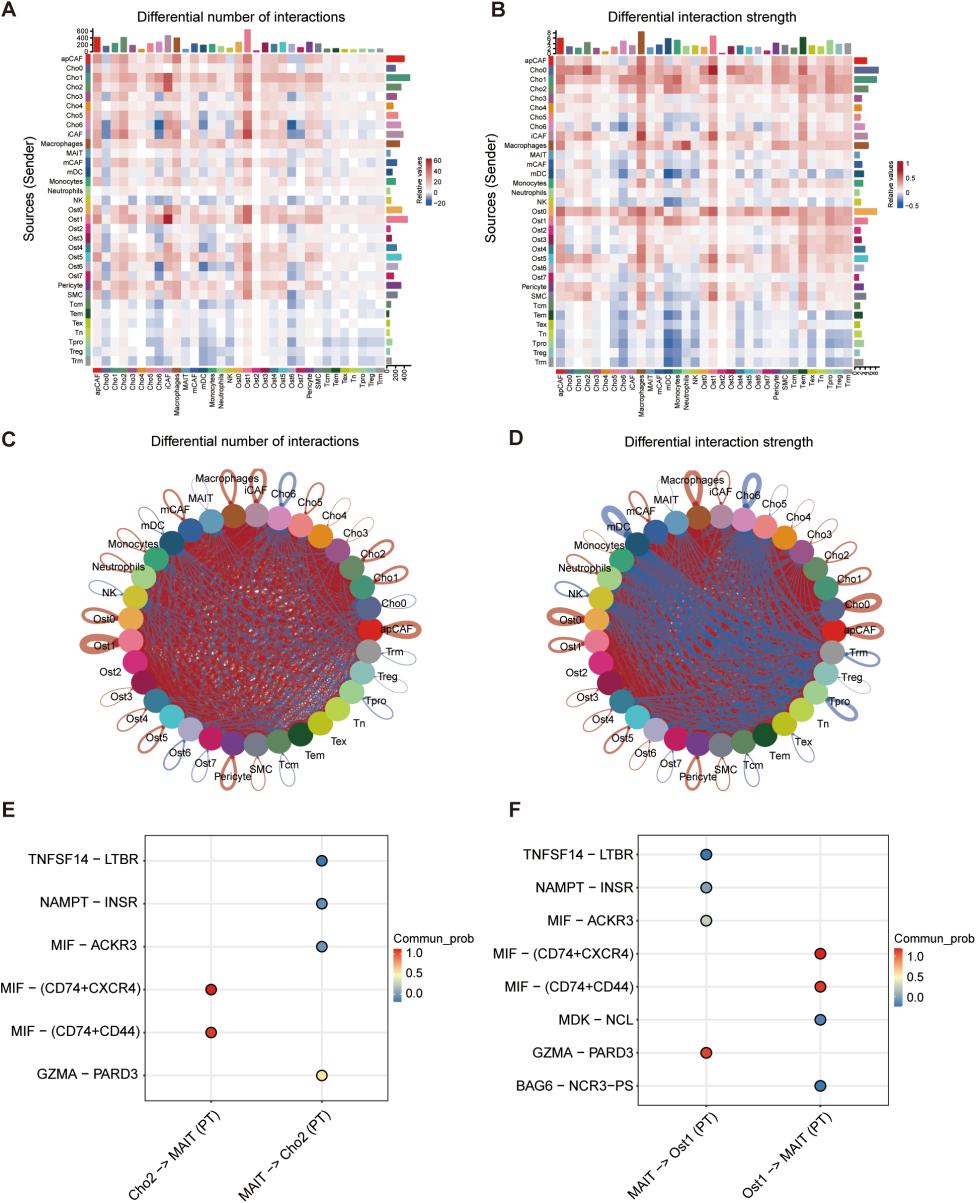
Cell-cell communication analysis between cell subtypes. **(A)** Heatmap showing differential number of predicted ligand-receptor interactions between cell types in PT versus normal tissue. **(B)** Heatmap showing differential interaction strength between cell types based on communication probability. **(C, D)** Circle plots illustrating overall patterns of altered cell-cell communication in PT compared to normal tissues, based on number of interactions **(C)** and interaction strength **(D)**. **(E, F)** Top predicted ligand-receptor pairs mediating interactions between specific cell pairs, including Cho2-MAIT and MAIT-Cho2 **(E)**, and MAIT-Ost1 and Ost1-MAIT **(F)**. Dot color indicates communication probability.

Intercellular communication within the TME is critical for shaping immune responses, maintaining tissue homeostasis, and driving tumor progression (28, 29). Herein, we proposed that malignant osteoblast subpopulations (Ost_1) and chondroblast subpopulations (Cho_2) drive osteosarcoma progression through immune evasion mechanisms mediated by specific ligand-receptor interactions. To characterize these interactions, we employed the CellChat algorithm to profile cell-cell communication networks between malignant cells and surrounding stromal/immune populations in osteosarcoma tissues. Comparative analysis with matched adjacent normal tissues revealed higher interaction frequency and signaling strength in tumor samples, reflecting an intensified communication landscape associated with malignancy (**Figures 6A, B**). Notably, T cell subsets demonstrated enhanced interaction with Ost_1 and Cho_2 cells in tumor tissues compared to normal tissues (**Figures 6C, D**; **Supplementary Figures S4A, B**). The macrophage migration inhibitory factor (MIF) signaling pathway emerged as a key mediator, with MIF-CD74+CD44 and MIF-CD74+CXCR4 axes prominently enriched in immune communication between malignant cells (Ost1 and Cho_2) and T cells (**Figures 6E, F**; **Supplementary Figures S4C, D**). This pathway has been previously linked to Treg accumulation in the TME and PD-L1 upregulation, both of which suppress effector T cell function and confer resistance to immune checkpoint inhibitors (30, 31). Collectively, these findings highlight a MIF-driven communication axis between malignant osteosarcoma cells and T cells that may underpin immune evasion and therapeutic resistance mechanisms.

### 3.7 Mitophagy-pyruvate metabolism coupling drives metabolic adaptation in malignant osteosarcoma cell states

**Figure 7:**
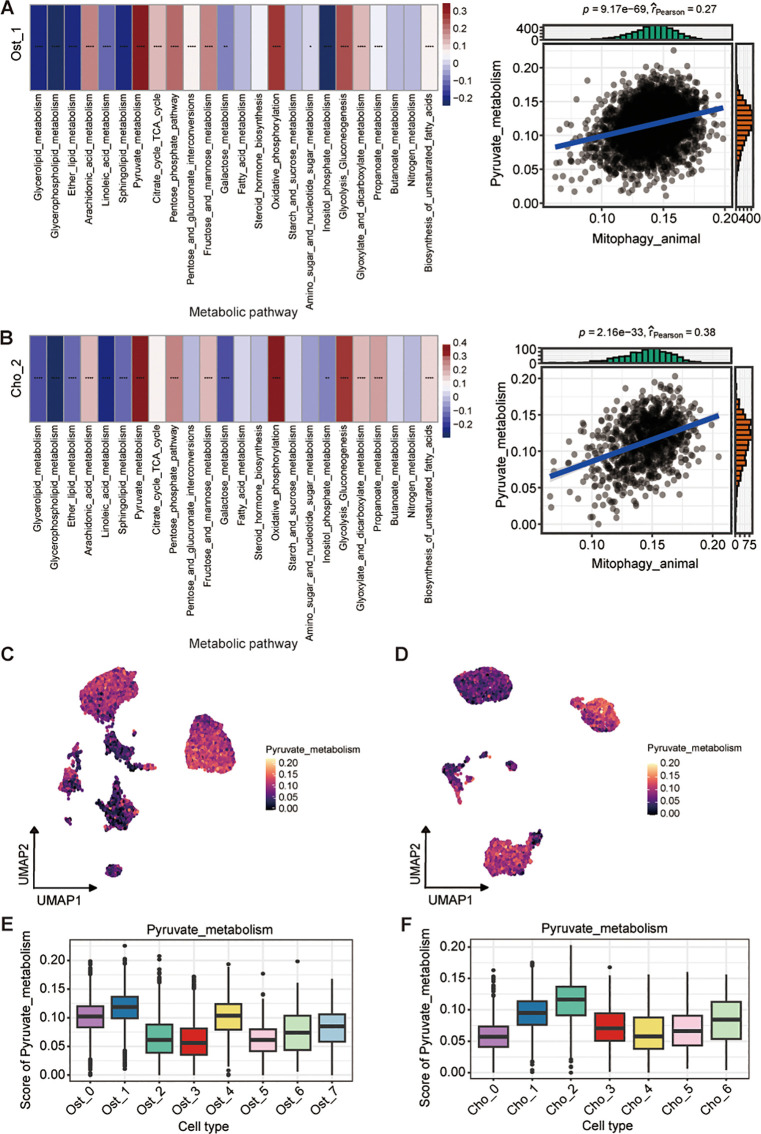
Association between mitophagy and pyruvate metabolism in osteoblast and chondroblast subpopulations. **(A, B)** Correlation between mitophagy and metabolic pathway activity in Ost_1 **(A)** and Cho_2 **(B)** clusters. Heatmaps show pathway scores across cells; (left; Wilcoxon rank-sum test). Scatter plots show positive Pearson correlations between mitophagy and pyruvate metabolism activity (blue line, linear fit; r and *P*-values indicated) (right). **(C, D)** UMAP plots showing distribution of pyruvate metabolism scores in osteoblast **(C)** and chondroblast **(D)** populations. **(E, F)** Boxplots of pyruvate metabolism scores across osteoblast clusters **(E)** and chondroblast clusters **(F)**. *P < 0.05; **P < 0.01; ***P < 0.001; ****P < 0.0001.

To investigate the role of mitophagy in metabolic reprogramming of osteosarcoma, we analyzed correlations between mitophagy activity and metabolic programs across Ost_1 and Cho_2 subpopulations. Both clusters demonstrated significant positive correlations between mitophagy and pyruvate metabolism, indicating functional coordination between mitochondrial quality control and metabolic adaptation (**Figures 7A, B**). To confirm this association, we analyzed bulk RNA sequencing data from the TARGET-OS cohort as an independent validation dataset. The results mirrored our single-cell findings that mitophagy activity remained significantly correlated with increased pyruvate metabolism, reinforcing the robustness of this relationship across data types and patient samples (**Supplementary Figure S5A**). We further explored whether this metabolic feature was specific to certain malignant cell states. Among all subpopulations examined, Ost_1 and Cho_2 clusters consistently exhibited the highest levels of pyruvate metabolic activity (**Figures 7C-F**), suggesting these malignant cell states uniquely integrate mitophagy and pyruvate metabolism to support tumor progression through coordinated metabolic rewiring.

### 3.8 GABARAP-mediated mitophagy drives chemoresistance and poor prognosis in osteosarcoma

**Figure 8:**
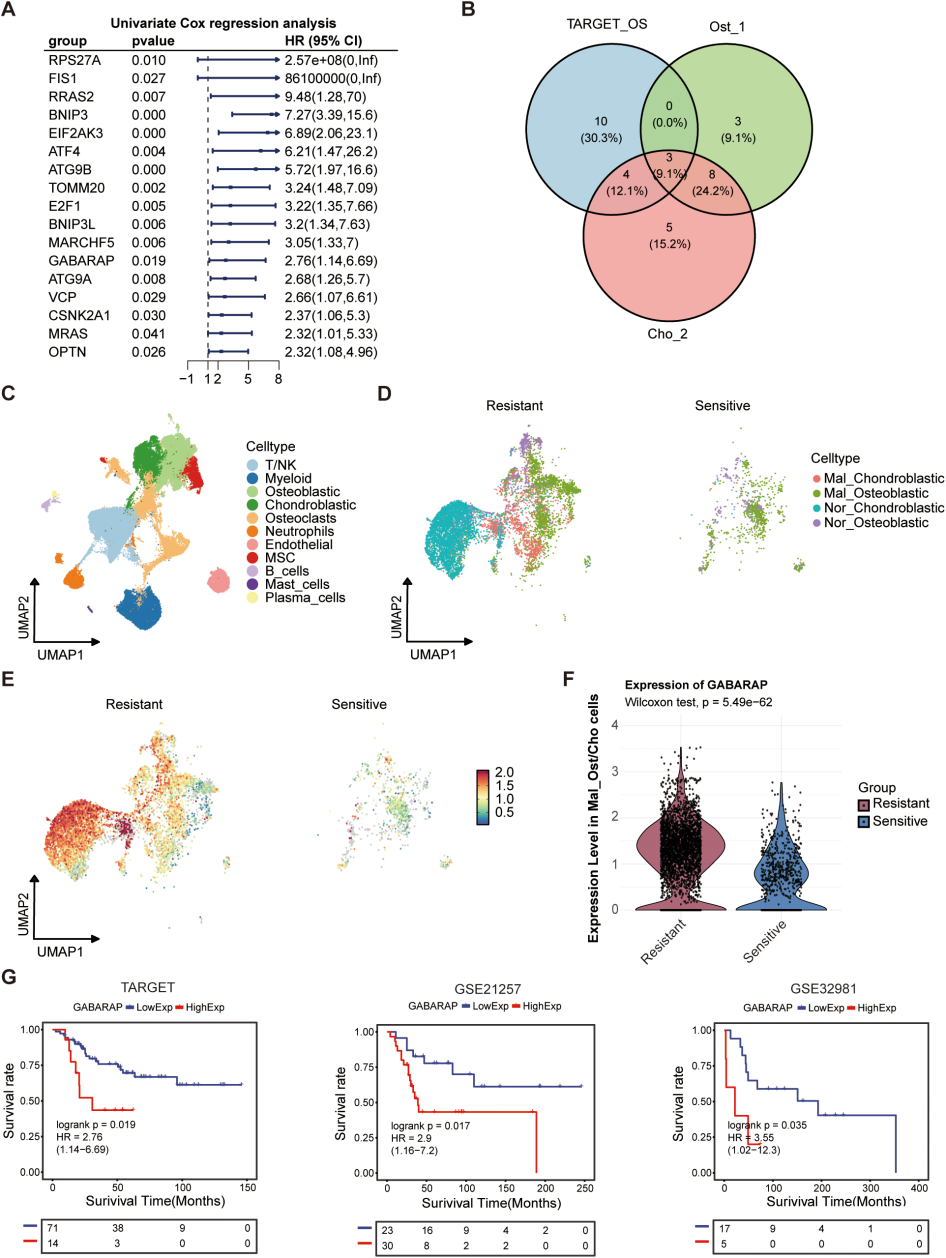
Prognostic significance and therapeutic association of mitophagy-related genes in osteosarcoma. **(A)** Univariate Cox regression analysis of autophagy-related genes in the TARGET osteosarcoma cohort. Hazard ratios (HRs), 95% confidence intervals (CIs), and P-values were shown. **(B)** Venn diagram showing overlapping prognostic autophagy-related genes among TARGET cohort, and osteoblast (Ost_1) and chondroblast (Cho_2) subpopulations from single-cell data. **(C)** UMAP plot of integrated osteosarcoma dataset form GitHub, annotated by major cell types. **(D)** Distribution of malignant (Mal_Ost/Cho) and normal (Nor_Ost/Cho) cells in chemo-resistant and chemo-sensitive patient groups, based on single-cell clustering. **(E)** GABARAP expression patterns in Ost/Cho subpopulations among chemo-resistant and chemo-sensitive patient groups. **(F)** Violin plots showing expression levels of GABARAP in malignant Ost/Cho subtypes among resistant and sensitive groups. **(G)** Kaplan-Meier survival analysis of GABARAP expression in three osteosarcoma cohorts: TARGET, GSE21257, and GSE32981.

Considering that mitophagy poses a significant role in shaping the prognosis of osteosarcoma patients, we performed univariate Cox regression analysis on bulk RNA-seq data from osteosarcoma cohorts. This analysis identified 17 mitophagy-related genes significantly associated with overall survival, suggesting a strong link between mitochondrial quality control and disease progression (**Figure 8A**). Subsequent scRNA-seq analysis of malignant subpopulations (Ost_1 and Cho_2) revealed three key genes (GABARAP, BNIP3, EIF2AK3) consistently overexpressed in tumor cells and linked to poor outcomes (**Figure 8B**). In order to investigate which is the most important gene in this progression, we constructed another atlas by integrating publicly available osteosarcoma scRNA-seq data (https://github.com/zhengxj1) with chemotherapy response information. Following dimensionality reduction, clustering, and batch effect correction, cells were assigned to 11 distinct clusters (**Figure 8C**). Each subgroup exhibited unique transcriptional signatures and pathway enrichment patterns (**Supplementary Figure S6A**), indicative of diverse functional roles. GO enrichment analysis revealed subgroup-specific characteristics: T/NK cells displayed cytolytic activity; osteoblastic cells showed enrichment for cadherin-mediated cell-cell adhesion and adherens junction organization; chondroblastic cells were enriched for regulation of fear response behavior and extracellular matrix organization (**Supplementary Figure S6A**). The major osteoblastic and chondroblastic (Ost/Cho) subpopulations were subsequently extracted and subclustered into 10 subsets (Osteoblastic_0-4/Chondroblastic_0-4) (**Supplementary Figure S6B**). Based on their functional implications in osteosarcoma malignancy, these 10 subsets were re-clustered into 4 populations (Mal_Ost/Cho and Nor_Ost/Cho) (**Supplementary Figure S7C**). Moreover, we performed unsupervised clustering of T/NK cells and annotated eight major subsets (**Supplementary Figures S6D, E**). Cell abundance analysis revealed an evident decrease in cytotoxic T lymphocytes (CTLs) and NKT cells, alongside Treg enrichment, in the resistant versus sensitive group (**Supplementary Figure S6F**), suggesting an immunosuppressive tumor microenvironment underlying chemoresistance. Correlation analyses further demonstrated that Mal_Ost/Cho abundance was negatively associated with CTLs, CD4^+^ naïve T cells, and CD4^+^Tm (**Supplementary Figure S6G**). And CTL proportions progressively declined as Mal_Ost/Cho fractions increased (**Supplementary Figure S6H**), supporting the above findings that Mal_Ost/Cho as tightly linked to poor prognosis and an immunosuppressive microenvironment in osteosarcoma.

Subsequently, we stratified Mal_Ost/Cho cells into chemo-sensitive and chemo-resistant groups to investigate the expression of key genes (**Figure 8D**). The results showed that the expression of GABARAP and BNIP3 in the chemo-resistant group was higher than that in the chemo-sensitive group (**Figures 8E, F**; **Supplementary Figures S7A, B**). However, the EIF2AK3 was lowly expressed in chemo-resistant tissues compared to the sensitive group (**Supplementary Figures S7C, D**). These findings indicated that GABARAP and BNIP3 may play an essential role in mediating chemotherapy resistance. Then, we further assessed the prognostic value of GABARAP and BNIP3 in three independent osteosarcoma cohorts, TARGET-OS, GSE21257, and GSE32981, respectively. Kaplan-Meier survival analysis revealed that high expression of GABARAP was associated with poor patient prognosis, whereas BNIP3 did not show a statistically significant association in the bulk RNA-seq validation using the GSE21257 dataset (**Figure 8G**; **Supplementary Figure S7E**). This consistent association underscored the potential of GABARAP as a robust predictor of clinical outcomes in osteosarcoma patients.

### 3.9 GABARAP defines spatial metabolic niches and immune-evading tumor states in osteosarcoma

**Figure 9:**
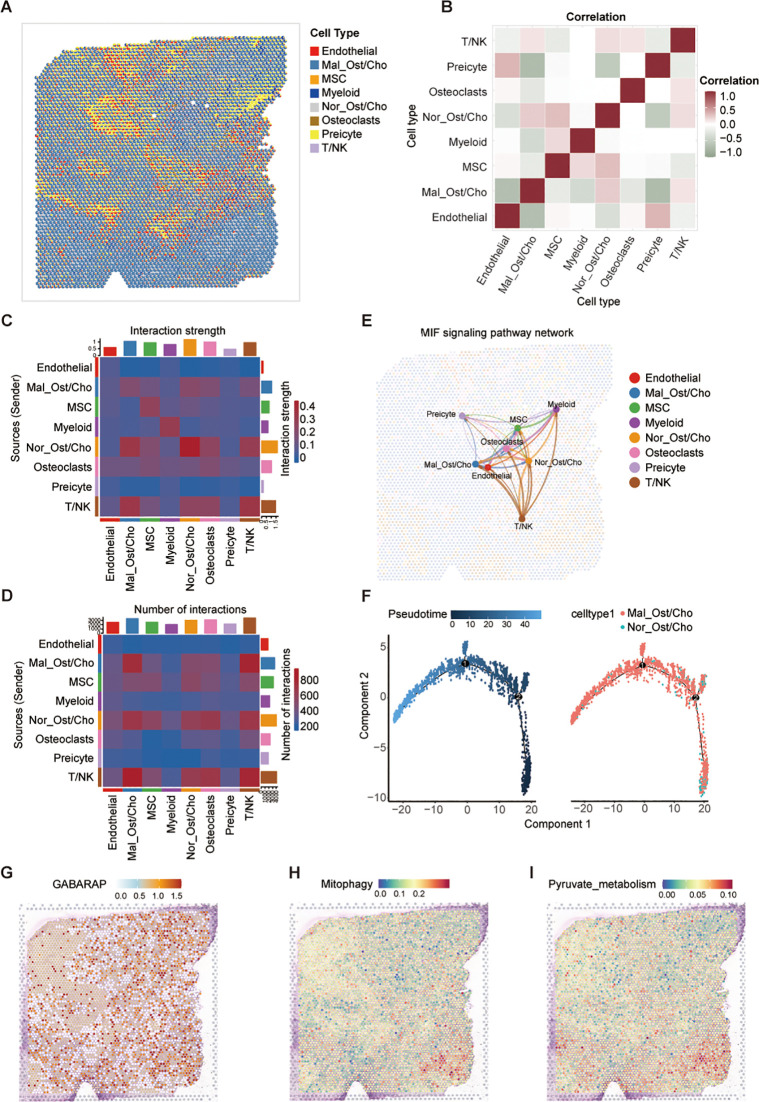
Spatial transcriptomic analysis reveals metabolic features and intercellular communication in osteosarcoma. **(A)** Spatial mapping of major cell types in osteosarcoma tissue using the CARD algorithm for cell-type deconvolution based on spatial transcriptomic data. **(B)** Correlation matrix of cell-type co-localization, showing spatial proximity between annotated cell populations. **(C, D)** Heatmaps showing predicted cell-cell communication patterns: interaction strength **(C)** and number of interactions **(D)** among different cell types, inferred from spatially resolved transcriptomes. **(E)** MIF signaling pathway network illustrating directional interactions between spatially localized cell types; edge thickness indicates communication strength. **(F)** Pseudotime trajectory analysis of Mal_Ost/Cho and Nor_Ost/Cho cells performed using Monocle2, colored by pseudotime (left) and cell type (right). **(G-I)** Spatial activity maps showing expression of GABARAP **(G)**, mitophagy pathway activity **(H)**, and pyruvate metabolism scores **(I)** across the tissue.

To elucidate the spatial distribution and functional implications of GABARAP, mitophagy, and pyruvate metabolism in osteosarcoma, we performed spatial transcriptomic profiling of primary osteosarcoma TME. Integration with previously acquired scRNA-seq data enhanced cell type annotation within spatially resolved transcriptomic maps, despite inter-dataset heterogeneity in cellular composition. In order to address the limited spatial resolution of standard spatial transcriptomics, we implemented the CARD deconvolution algorithm to estimate subspot-level cell composition within tissue sections. This approach identified 11 distinct spatial clusters, which were annotated into seven cell types: Ost/Cho cells, MSCs, myeloid cells, osteoclasts, pericytes, and T/NK cells (**Figure 9A**; **Supplementary Figures S8A, B**). Notably, Ost/Cho cells exhibited bimodal classification into malignant (Mal_Ost/Cho) and normal (Nor_Ost/Cho) subpopulations (**Figure 9A**). We observed that multiple cell types inferred by CARD show spatially co-localization patterns (**Figure 9B**). Specially, Mal_Ost/Cho cells mostly co-localized spatially with T/NK and Nor_Ost/Cho cells, supporting the role of the former interaction in forming the immunosuppressive microenvironment in osteosarcoma. Complementing this, cell-cell communication analysis demonstrated enhanced signaling through the MIF pathway between Mal_Ost/Cho and T/NK cells (**Figures 9C-E**; **Supplementary Figure S8C**), validating the MIF-mediated intercellular communication axis enables immune evasion in osteosarcoma.

Consequently, we performed spatial pseudotime analysis to trace the developmental trajectory of malignant Ost/Cho cells. We observed a clear progression from normal to malignant states, with dynamic changes in gene expression along this path (**Figure 9F**; **Supplementary Figures S8D, E**), identifying key regulators of tumor evolution. Besides, we found that GABARAP expression exhibited widespread tumor distribution with pronounced enrichment in malignant Ost/Cho cells (**Figure 9G**). To better understand the potential link between GABARAP and key metabolic processes, we examined the spatial localization of mitophagy and pyruvate metabolism-related genes. Spatial co-localization analysis demonstrated that GABARAP expression aligned with these metabolic hotspots and both pathways showed strong spatial overlap (**Figures 9H, I**), particularly in regions densely populated by malignant Ost/Cho cells. These findings collectively underscored the dual role of GABARAP in promoting osteosarcoma progression through metabolic reprogramming and immune evasion mechanisms, as evidenced by its spatial association with critical metabolic pathways and immunosuppressive TME features.

### 3.10 GABARAP drives mitophagy and pyruvate metabolic reprogramming to promote osteosarcoma progression

**Figure 10:**
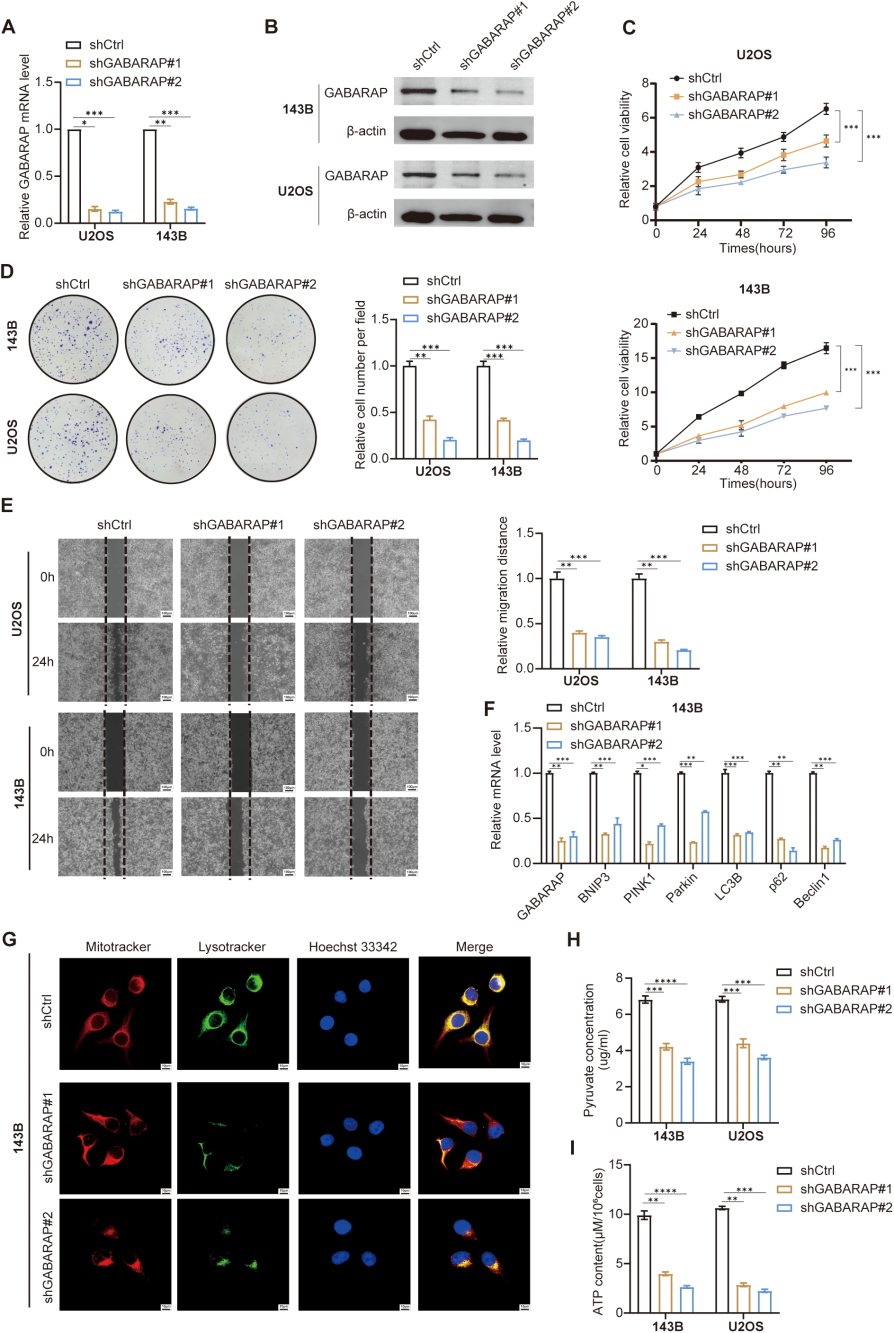
GABARAP knockdown impairs osteosarcoma cell proliferation, migration, mitophagy, and energy metabolism. **(A)** qRT-PCR analysis of GABARAP mRNA expression in U2OS and 143B cells transduced with control shRNA (shCtrl) or two independent GABARAP-silenced shRNAs (shGABARAP#1, shGABARAP#2). **(B)** Western blot confirming reduced GABARAP protein levels following knockdown in both cell lines. **(C)** Cell proliferation curves assessed by CCK-8 assay at indicated time points (0, 24, 48, 72, 96 h). **(D)** Colony formation assays demonstrating reduced clonogenicity upon GABARAP silencing; representative images (left) and quantification (right). **(E)** Wound healing assays showing impaired cell migration at 24 h post-scratch; quantified by relative migration distance. Scale bar, 100 μm. **(F)** qRT-PCR analysis of mitophagy-related genes (BNIP3, PINK1, Parkin, LC3B, p62, Beclin1) in 143B cells following GABARAP knockdown. **(G)** Confocal microscopy of 143B cells stained with MitoTracker (red), LysoTracker (green), and Hoechst 33342 (blue); merged images show disrupted mitochondria-lysosome colocalization in GABARAP-deficient cells. Scale bar, 100 μm. **(H)** Quantification of intracellular pyruvate levels in 143B and U2OS cells. **(I)** Measurement of ATP content per 10^6^ cells in both cell lines. Data are presented as mean ± s.d. from at least three independent experiments. Statistical comparisons were performed using two-tailed unpaired Student’s t-test. *P < 0.05, **P < 0.01, ***P < 0.001, ****P < 0.0001.

To investigate the functional significance of GABARAP in osteosarcoma, we generated stable knockdown models in 143B and U2OS cells using lentivirus-mediated shRNAs (shGABARAP#1 and shGABARAP#2). RT-qPCR validation confirmed effective gene silencing (**Figure 10A**). Western blot analysis further validated protein-level depletion (**Figure 10B**), prompting retention of both shRNA constructs for subsequent functional studies. CCK-8-based proliferation assays revealed pronounced reduced cell viability over 96-hour observation periods in GABARAP-deficient cells (**Figure 10C**). Colony formation assays corroborated these findings, showing marked reductions in both colony quantity and size (**Figure 10D**), indicating the essential role of GABARAP in osteosarcoma cell proliferation. Wound healing assays demonstrated impaired migratory capacity in GABARAP-silenced cells (**Figure 10E**), demonstrating that GABARAP may contribute to the migratory potential of osteosarcoma cells.

Considering the effect of GABARAP on mitophagy in osteosarcoma, we next explored the expression of several key mitophagy-associated genes, including BNIP3, PINK1, Parkin, LC3B, p62, and Beclin1, which were significantly downregulated in GABARAP knockdown cells (**Figure 10F**; **Supplementary Figure S9A**). To assess mitophagy activity, cells were stained with MitoTracker and LysoTracker to label mitochondria and lysosomes, respectively. Confocal imaging revealed reduced LysoTracker intensity in GABARAP-silenced cells (**Figure 10G**; **Supplementary Figure S9B**), indicating compromised mitophagy activity. Next, we further evaluate the pyruvate metabolic consequences of GABARAP knockdown through detecting the intracellular levels of pyruvate and ATP. Metabolic assessments demonstrated decreased intracellular pyruvate and ATP levels in GABARAP-depleted cells (**Figures 10H, I**), reflecting disrupted pyruvate metabolism. Collectively, these findings establish GABARAP as a central regulator of osteosarcoma cell proliferation, migration, and metabolic fitness, operating through mitophagy modulation and pyruvate metabolic reprogramming.

## 4 Discussion

Osteosarcoma remains a highly aggressive malignancy with unfavorable clinical outcomes despite advancements in surgical and chemotherapeutic modalities, particularly in managing advanced and recurrent disease (32–34). Metastatic dissemination, chemoresistance, and disease recurrence collectively drive poor prognoses, yet the pathogenic mechanisms governing these processes remain incompletely elucidated (35). This study employed an integrated analytical framework combining scRNA-seq, spatial transcriptomics, and bulk RNA-seq to identify aggressive osteosarcoma subpopulations (Ost_1/Cho_2) characterized by mitophagy-pyruvate metabolic crosstalk. Functional validation established GABARAP as a central oncogenic regulator orchestrating synergistic mitophagy flux and pyruvate metabolic reprogramming, thereby identifying GABARAP as a promising therapeutic target for osteosarcoma progression.

In our study, we defined five fibroblast subtypes and found striking dominance of mCAFs alongside severe contraction of iCAFs in tumor tissues. These observations reinforce and extend recent single-cell atlases in osteosarcoma, where matrix-driven CAFs intimately engage endothelial and stromal networks (20). The upregulation of POSTN in our tumor-derived mCAF cluster resonates with reports that POSTN^+^ CAFs as potent immune response barriers at specific tumor locations, as they hinder effective T-cell infiltration and decrease the efficacy of immunotherapy (36). Our diminished iCAF compartment may reflect tumor spatial constraints or sampling bias; notably, osteosarcoma exosome‐induced priming of fibroblasts toward cytokine-rich phenotypes suggests that iCAF programs might manifest preferentially in premetastatic or peritumoral niches rather than in bulk primary tumor samples (37). The presence of MHC-II-high apCAFs mirrors immunoregulatory fibroblast states described across cancers and suggests stromal participation in antigen presentation and T-cell trafficking in osteosarcoma, with implications for immunotherapy response prediction (38). Concurrent recovery of pericytes and SMCs, together with contractile mCAFs, supports a vascular-stromal axis that shapes perfusion, stiffness gradients, and barrier properties, potentially influencing drug delivery and immune infiltration (39).

Our myeloid profiling redefines osteosarcoma as a macrophage-dominated ecosystem with a pronounced M2-skewed transcriptional program, consistent with contemporary mechanistic and translational literature (40). The axis of CCL2/CCR2-mediated monocyte recruitment and polarization to M2-like TAMs has been causally linked to metastatic seeding in osteosarcoma models, a finding consistent with our inference of enhanced monocyte-to-macrophage differentiation within tumors (41, 42). The predominance of an M2 phenotype in our data echoes the central dependence of M2 TAMs on CSF-1/CSF-1R signaling: blockade of CSF-1R (e.g. with pexidartinib or PLX3397) is known to reprogram or deplete TAMs, modulate the tumor immune microenvironment, and inhibit sarcoma growth in preclinical models, lending translational rationale to our findings (43). Further, the pro-tumor cytokines and chemokines we detect (TNF, CCL20 and MMP19) are coherent with models in which osteosarcoma cell-derived exosomes or TIM-3-related cargo drive macrophages toward M2 states, thereby amplifying invasive and immunosuppressive circuits; experimental disruption of such signaling impairs metastatic competence in osteosarcoma models (44). Clinically, our results reinforce the paradigm that M1-biased macrophage signatures are associated with favorable prognosis, whereas high burdens of M2/TAM subsets (e.g. CD163^+^ or EPOR^+^CD163^+^ TAMs) predict aggressive disease and metastatic potential in osteosarcoma patients (45, 46).

In this study, we identified a heterogeneous but functionally constrained T/NK ecosystem in osteosarcoma, where enrichment of FOXP3^+^ Tregs and exhausted CD8^+^ T cells paralleled recent single-cell atlases that describe pervasive T-cell dysfunction and regulatory dominance (47). The prominence of Tregs in tumors supports prior evidence that their accumulation suppresses CTL responses and associates with poor prognosis, suggesting that targeting this subset may be critical for restoring antitumor immunity (48). Our CellChat analysis further revealed intensified malignant cell-T/NK communication, with MIF-CD74-CD44/CXCR4 emerging as a dominant axis, consistent with reports that MIF signaling promotes immunosuppressive crosstalk, PD-L1 induction, and metastatic potential (49). Importantly, tumor-derived MIF has been shown to drive NF-κB activation, proliferation, and metastasis, while pharmacologic destabilization or neutralization of MIF suppresses tumor progression and enhances responses to PD-1 blockade, supporting its translational relevance in osteosarcoma (50). The observation that resistant tumors exhibited reduced CTLs and NKT cells but enriched Tregs mirrors findings that chemotherapy reshapes the osteosarcoma immune landscape yet leaves exhaustion and Treg circuits entrenched (48, 51). Moreover, spatial co-localization of malignant Ost/Cho with T/NK cells and enhanced MIF signaling in our data parallels recent evidence that post-chemotherapy osteosarcoma retains suppressive ligand-receptor interactions and metabolic drivers of PD-L1 upregulation, further consolidating MIF as a central mediator of immune evasion (49, 52).

Our findings demonstrated that mitophagy closely aligns with pyruvate metabolism in malignant osteosarcoma states, supporting the concept that mitochondrial quality control and metabolic reprogramming operate in concert to sustain tumor progression. Recent reports further reinforce this notion. For example, the FoxG1-BNIP3 axis has been shown to promote mitophagy and confer cisplatin resistance, indicating that targeting mitophagy can resensitize osteosarcoma cells to chemotherapy (11). Similarly, hypoxia activates a FOXO3a/HSP90-FUNDC1 pathway that enhances mitochondrial turnover and reduces cisplatin-induced apoptosis, underscoring the role of mitophagy as a protective mechanism under stress (10). At the transcriptomic level, mitophagy-related genes have been identified as drivers of metastasis and prognostic markers in osteosarcoma, highlighting their contribution to malignant potential (15). On the metabolic side, osteosarcoma cells typically exhibit glycolysis-biased pyruvate metabolism through PKM2, LDHA, and lactate transporters, processes that not only support proliferation but also remodel the tumor microenvironment and impair immune function (7, 53). Notably, pyruvate dehydrogenase kinase 1 (PDK1) has emerged as a key regulator of this metabolic shift, and its inhibition restores oxidative metabolism and suppresses stemness features (12). Together, we may propose that therapeutic strategies combining mitophagy blockade with metabolic interventions that force pyruvate toward mitochondrial oxidation (e.g., PDK1 inhibitors or lactate transport blockade) hold promise for disrupting tumor plasticity and overcoming chemoresistance in osteosarcoma.

In this study, GABARAP emerged as a pivotal factor in osteosarcoma progression through its dual roles in mitophagy and metabolic regulation. Consistent with recent findings that autophagy is indispensable for osteosarcoma growth and therapy adaptation (54), GABARAP appears to coordinate mitochondrial quality control with metabolic resilience. Mechanistically, GABARAP is known to scaffold the FLCN-FNIP complex and regulate TFEB, linking autophagy to lysosomal biogenesis and nutrient sensing (55). In other cancers, its role is context-dependent: GABARAP promotes tumor aggressiveness and poor survival in colorectal carcinoma (56), whereas in breast cancer it restrains epithelial-mesenchymal transition and invasion (57). Moreover, loss of GABARAP compromises immunogenic cell death in multiple myeloma, reducing therapy efficacy (58), while in lung cancer, activation of a GABARAP-NIX axis induces selective mitophagy and radiosensitization (59). These reports highlight GABARAP’s pleiotropic but central involvement in tumor biology. The connection between GABARAP and mitophagy is well established, with BNIP3/NIX and FUNDC1 serving as receptors that engage LC3/GABARAP proteins during mitochondrial clearance (60). Structural reconstitution studies further confirm GABARAP as essential for autophagosome maturation in receptor-driven mitophagy (61). Importantly, this mitophagic activity intersects with pyruvate metabolism, as removal of damaged mitochondria preserves PDH-TCA coupling and sustains oxidative phosphorylation. Such metabolic buffering prevents forced glycolytic routing of pyruvate, maintaining ATP generation under stress (55, 62). In osteosarcoma, where chemoresistance often correlates with metabolic rewiring, these functions provide a plausible mechanism for GABARAP-mediated therapy resistance. The convergence of autophagy, mitophagy, and pyruvate metabolism positions GABARAP as a potential therapeutic target. Given its diverse roles across cancers, strategies aimed at modulating GABARAP activity, either inhibiting its tumor-supportive functions or exploiting its capacity to induce lethal stress responses, may offer translational opportunities in osteosarcoma and other malignancies.

Despite the strengths of this study, several limitations should be acknowledged. The normal group contained only 2 samples, which limited the number of samples available for statistical analysis when comparing cell-type proportions with the PT group. The number of spatial transcriptomic osteosarcoma samples remained limited, and additional data were needed to validate these findings across broader cohorts. Furthermore, our conclusions regarding mitophagy’s role in the TME were largely based on computational inferences and require further experimental confirmation. Finally, the impact of GABARAP on osteosarcoma progression has yet to be validated *in vivo* or in clinical settings.

## 5 Conclusion

Osteosarcoma is a highly aggressive bone cancer with poor prognosis. scRNA-seq identified two malignant subtypes (Ost_1, Cho_2) linked to poor prognosis, exhibiting upregulated mitophagy and pyruvate metabolic rewiring. GABARAP was identified as a central regulator, enriched in metabolic hotspots and immune-evasive niches. Its overexpression enhances mitophagy, metabolic adaptation, proliferation, migration, and chemoresistance. These findings highlight GABARAP as a pivotal driver of osteosarcoma malignancy and a promising therapeutic target linking mitochondrial quality control with metabolic reprogramming.

## Data Availability

The original contributions presented in the study are included in the article/**Supplementary Material**. Further inquiries can be directed to the corresponding author/s.

## Supplementary material

Supplementary Figure 1 Characterization of the landscapes for fibroblast. **(A)** UMAP of fibroblast subtypes. Pericytes; SMC: smooth muscle cells; mCAFs: matrix-producing cancer-associated fibroblasts; iCAFs: inflammatory cancer-associated fibroblasts; apCAFs: antigen-presenting cancer-associated fibroblasts. **(B)** Fibroblast subtype distribution in normal and tumor tissues. **(C)** Dot plot of representative marker gene expression across fibroblast subtypes. **(D)** Violin plots showing the normalized expression levels of five representative marker genes across the five fibroblast subsets. **(E)** Heatmap of DEGs across fibroblast clusters between normal and PT samples. **(F)** Fibroblast subtype frequencies across normal and PT groups. **(G)** Box plots show the percentage of five fibroblast cell types in normal and PT, respectively.

Supplementary Figure 2 A mitophagy-driven osteoblastic cell state initiates osteosarcoma progression. **(A-C)** UMAP plots showing sample origin **(A)**, group **(B)**, and inferred CNV scores **(C)**. **(D, E)** Bar plots showing proportions of malignant cells **(D)** and survival-associated profiles **(E)** across clusters. **(F)** Mitophagy pathway activity across osteoblast clusters. **(G)** CytoTRACE-inferred differentiation potential. **(H, I)** Monocle2-based pseudotime trajectory colored by cluster **(H)** and cell state **(I)**. **(J)** Branched heatmap of dynamic gene expression along fate decisions. **(K, L)** Bar plots show GO and KEGG enrichment of fate-specific gene sets.

Supplementary Figure 3 Transcriptional landscape and trajectory of chondroblast subpopulations. **(A-C)** UMAP plots showing sample origin **(A)**, group **(B)**, and CNV scores **(C)**. **(D, E)** Proportions of malignant cells **(D)** and survival-related states **(E)** across clusters. **(F)** Mitophagy activity scores among chondroblast clusters. **(G)** CytoTRACE prediction of differentiation potential. **(H, I)** Monocle 2-inferred pseudotime trajectory colored by cluster **(H)** and state **(I)**. **(J)** Heatmap of gene expression dynamics along fate branches., **(K, L)** GO and KEGG enrichment of branch-specific genes.

Supplementary Figure 4 Cell-cell communication patterns between malignant osteoblast/chondroblast clusters and immune cells in the TME. **(A, B)** Circle plots showing outgoing interactions from Cho_2 **(A)** and Ost_1 **(B)** to various immune and stromal cell types in primary tumor samples. Line thickness represents number of predicted ligand-receptor interactions. **(C)** Top predicted ligand-receptor pairs mediating interactions from Ost_1 to multiple immune cell types in tumor samples. **(D)** Ligand-receptor interactions from Cho_2 to the same immune subsets in the tumor microenvironment.

Supplementary Figure 5 Mitophagy is positively correlated with pyruvate metabolism in osteosarcoma. **(A)** Correlation between mitophagy and metabolic pathway activity in TARGET. Heatmaps show pathway scores across cells (left; Wilcoxon rank-sum test). Scatter plots show positive Pearson correlations between mitophagy and pyruvate metabolism activity (blue line, linear fit; r and *P*-values indicated) (right). **P* < 0.05; ***P* < 0.01; ****P* < 0.001; *****P *< 0.0001.

Supplementary Figure 6 Functional characterization and the interaction between malignant cells and T/NK cells in osteosarcoma. **(A)** Module analysis of gene expression programs across cell subsets using gene set variation and enrichment scores; top associated GO terms are shown. **(B)** Ost/Cho cell type composition in osteosarcoma samples. **(C)** UMAP plots showing distribution of Mal_Ost/Cho and Nor_Ost/Cho cells. **(D)** UMAP of T/NK cells in GitHub. CTLs: cytotoxic T lymphocytes. CD4^+^ naïve T cells. CD4^+^Tm: CD4^+^ memory T cells. NKT cells. Treg: regulatory T cells. CD8^+^ naïve T cells. Tfh: follicular helper T cell. CD8^+^Tm: CD8^+^ memory T cells. **(E)** T/NK cell subtypes distribution in chemo-sensitive and chemo-resistant tissues. **(F)** T/NK cell subtypes (GitHub) frequencies across normal and PT groups. **(G)** Correlation heatmap between Mal_Ost/Cho and T/NK cell subpopulations. The values are displayed on a color scale from blue (negative correlation) to red (positive correlation), with significant correlations marked by asterisks. **(H)** Pearson correlation analysis of CTL and Mal_Ost/Cho proportion. **P* < 0.05, ***P* < 0.01, ****P* < 0.001.

Supplementary Figure 7 Clinical relevance of key genes in osteosarcoma. **(A)** BNIP3 expression pattern in Ost/Cho subpopulations among chemo-resistant and chemo-sensitive groups. **(B)** Expression levels of BNIP3 in malignant Ost/Cho subtypes among resistant and sensitive groups. **(C)** EIF2AK3 expression pattern in Ost/Cho subpopulations among chemo-resistant and chemo-sensitive groups. **(D)** Expression levels of EIF2AK3 in malignant Ost/Cho subtypes among resistant and sensitive groups. **(E)** Kaplan-Meier survival analysis of BNIP3 expression in the TARGET, GSE21257, and GSE32981 osteosarcoma cohorts; *P*-values by log-rank test and HR.

Supplementary Figure 8 Spatial transcriptomic analysis reveals metabolic features and intercellular communication in osteosarcoma. **(A, B)** UMAP plot **(A)** and spatial mapping **(B)** of cell clusters identified using Seurat (clusters 0-10). **(C)** Network diagrams showing the number (left) and strength (right) of predicted ligand-receptor interactions among major cell populations in the spatial transcriptomic dataset. **(D)** Spatial visualization of pseudotime (top) and malignant vs. normal Ost/Cho cells distribution (bottom). **(E)** Branched heatmap showing expression dynamics of key genes across pseudotime-associated clusters.

Supplementary Figure 9 GABARAP knockdown impairs mitophagy and mitophagy-related gene expression in U2OS cells. **(A)** qRT-PCR analysis of GABARAP, BNIP3, PINK1, Parkin, LC3B, p62 and Beclin1 mRNA levels in U2OS cells after GABARAP knockdown. **(B)** Confocal microscopy images of U2OS cells labeled with MitoTracker (red), LysoTracker (green), and Hoechst 33342 (blue). Merged images show reduced colocalization of mitochondria and lysosomes in GABARAP-silenced cells (shGABARAP#1 and shGABARAP#2). Data are shown as mean ± s.d. from three independent experiments. **P *< 0.05, ***P* < 0.01, ****P* < 0.001.

Supplementary Table 1 Clinical characteristics of patients in the present study.

Supplementary Table 2 Marker of Cell type annotation.

Supplementary Table 3 Primer sequences of mitophagy-related gene.
