# Supplementary figures and images for: Integrated multi-omics reveals GABARAP-mediated mitophagy and pyruvate metabolism as key drivers of osteosarcoma progression
Source: Front Immunol. 2025 Nov 19;16:1680554. doi: 10.3389/fimmu.2025.1680554 (PMC12672450; doi:10.3389/fimmu.2025.1680554)

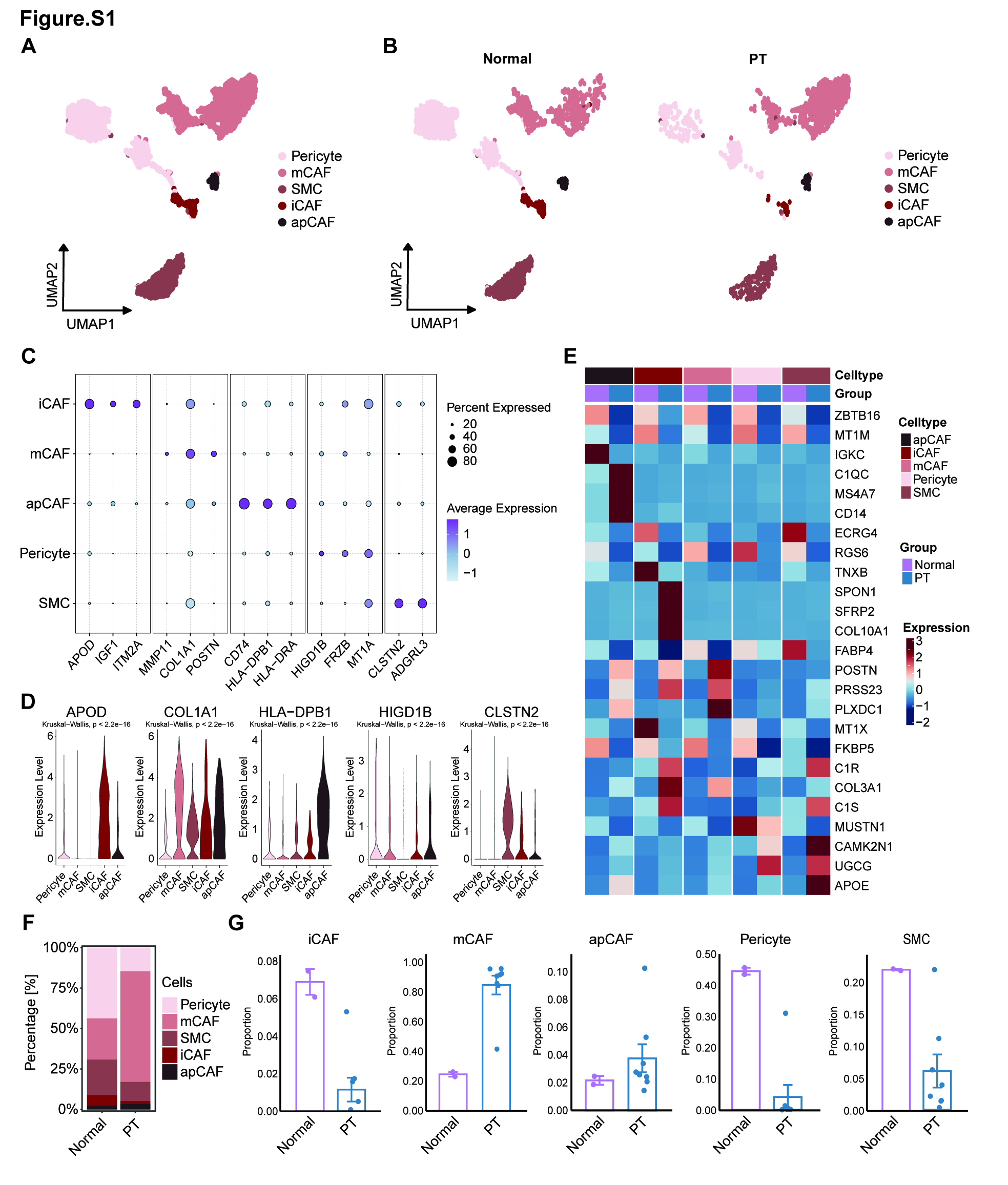

Supplement: Supplementary Figure 1 — Characterization of the landscapes for fibroblast. (A) UMAP of fibroblast subtypes. Pericytes; SMC: smooth muscle cells; mCAFs: matrix-producing cancer-associated fibroblasts; iCAFs: inflammatory cancer-associated fibroblasts; apCAFs: antigen-presenting cancer-associated fibroblasts. (B) Fibroblast subtype distribution in normal and tumor tissues. (C) Dot plot of representative marker gene expression across fibroblast subtypes. (D) Violin plots showing the normalized expression levels of five representative marker genes across the five fibroblast subsets. (E) Heatmap of DEGs across fibroblast clusters between normal and PT samples. (F) Fibroblast subtype frequencies across normal and PT groups. (G) Box plots show the percentage of five fibroblast cell types in normal and PT, respectively. [file Image1.tif]

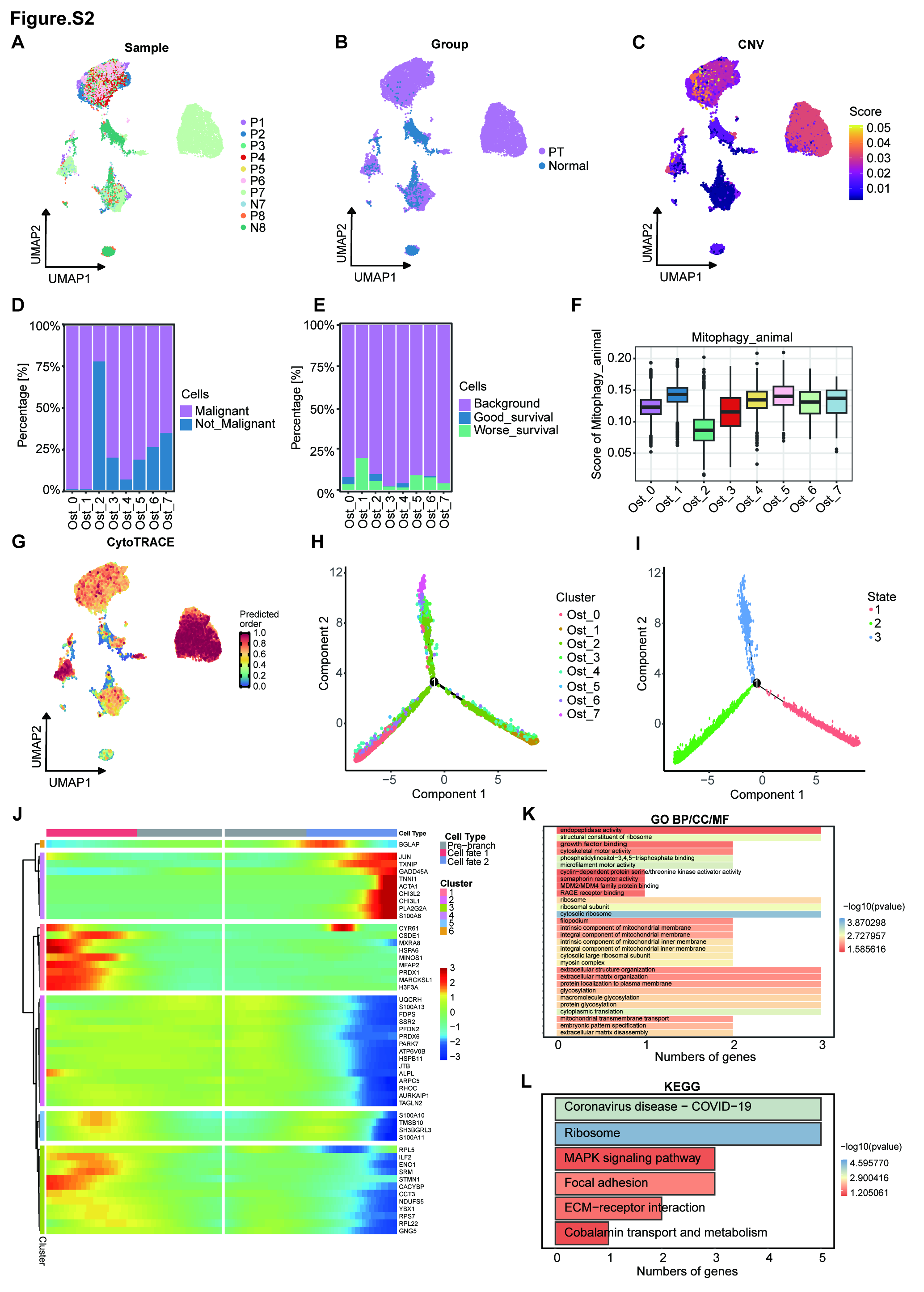

Supplement: Supplementary Figure 2 — A mitophagy-driven osteoblastic cell state initiates osteosarcoma progression. (A-C) UMAP plots showing sample origin (A), group (B), and inferred CNV scores (C). (D, E) Bar plots showing proportions of malignant cells (D) and survival-associated profiles (E) across clusters. (F) Mitophagy pathway activity across osteoblast clusters. (G) CytoTRACE-inferred differentiation potential. (H, I) Monocle2-based pseudotime trajectory colored by cluster (H) and cell state (I). (J) Branched heatmap of dynamic gene expression along fate decisions. (K, L) Bar plots show GO and KEGG enrichment of fate-specific gene sets. [file Image2.tif]

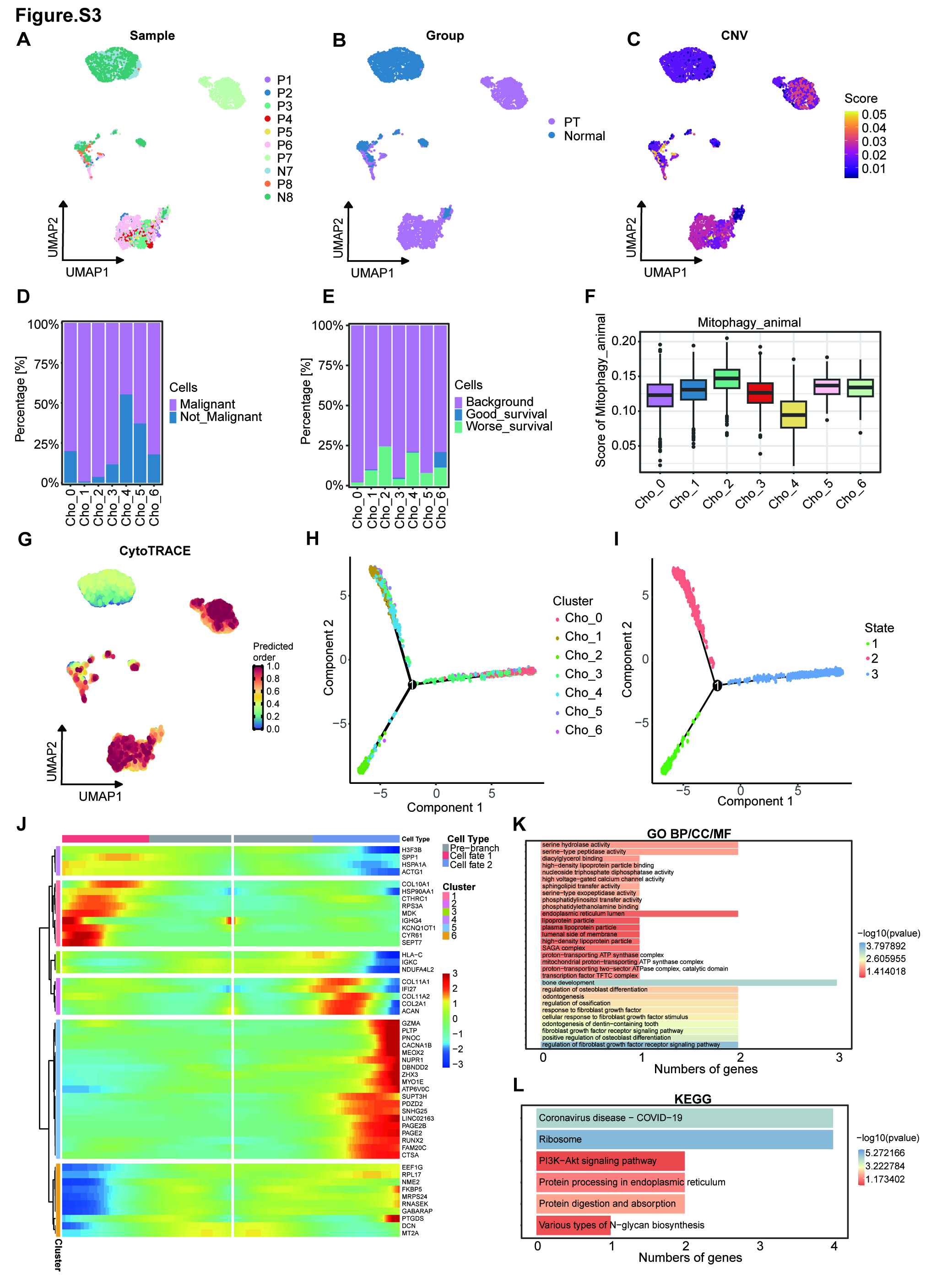

Supplement: Supplementary Figure 3 — Transcriptional landscape and trajectory of chondroblast subpopulations. (A-C) UMAP plots showing sample origin (A), group (B), and CNV scores (C). (D, E) Proportions of malignant cells (D) and survival-related states (E) across clusters. (F) Mitophagy activity scores among chondroblast clusters. (G) CytoTRACE prediction of differentiation potential. (H, I) Monocle 2-inferred pseudotime trajectory colored by cluster (H) and state (I). (J) Heatmap of gene expression dynamics along fate branches., (K, L) GO and KEGG enrichment of branch-specific genes. [file Image3.tif]

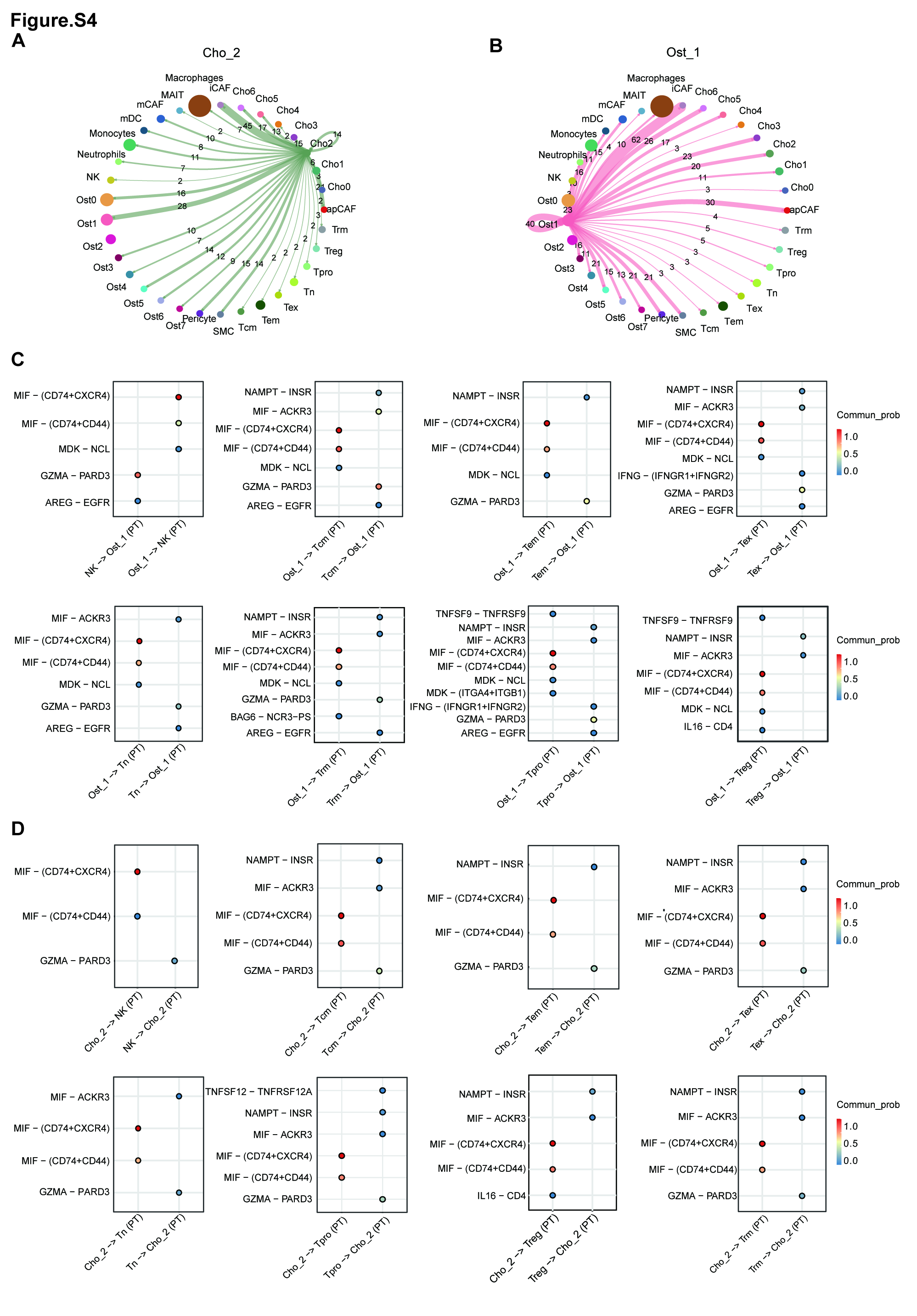

Supplement: Supplementary Figure 4 — Cell-cell communication patterns between malignant osteoblast/chondroblast clusters and immune cells in the TME. (A, B) Circle plots showing outgoing interactions from Cho_2 (A) and Ost_1 (B) to various immune and stromal cell types in primary tumor samples. Line thickness represents number of predicted ligand-receptor interactions. (C) Top predicted ligand-receptor pairs mediating interactions from Ost_1 to multiple immune cell types in tumor samples. (D) Ligand-receptor interactions from Cho_2 to the same immune subsets in the tumor microenvironment. [file Image4.tif]

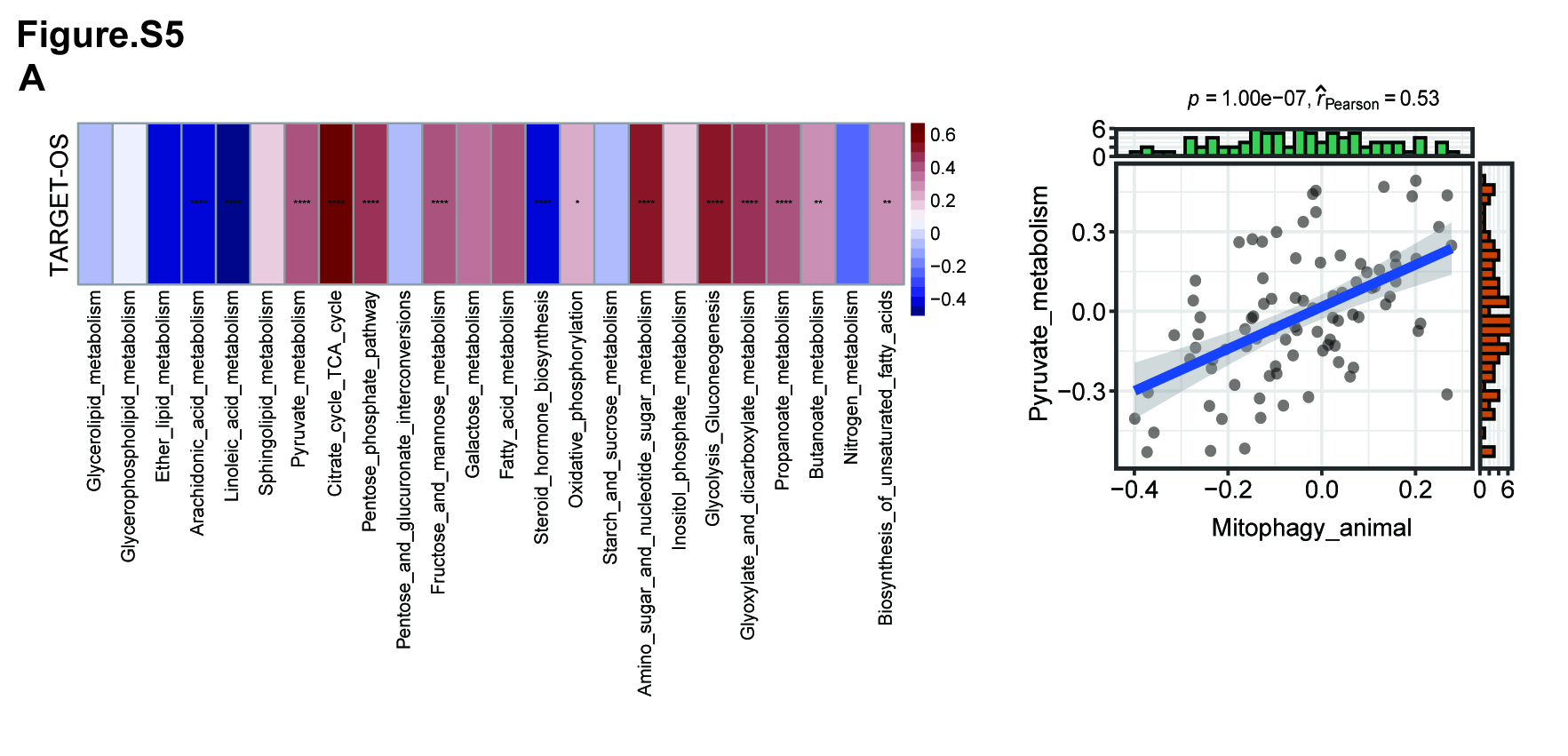

Supplement: Supplementary Figure 5 — Mitophagy is positively correlated with pyruvate metabolism in osteosarcoma. (A) Correlation between mitophagy and metabolic pathway activity in TARGET. Heatmaps show pathway scores across cells (left; Wilcoxon rank-sum test). Scatter plots show positive Pearson correlations between mitophagy and pyruvate metabolism activity (blue line, linear fit; r and P-values indicated) (right). *P < 0.05; **P < 0.01; ***P < 0.001; ****P < 0.0001. [file Image5.tif]

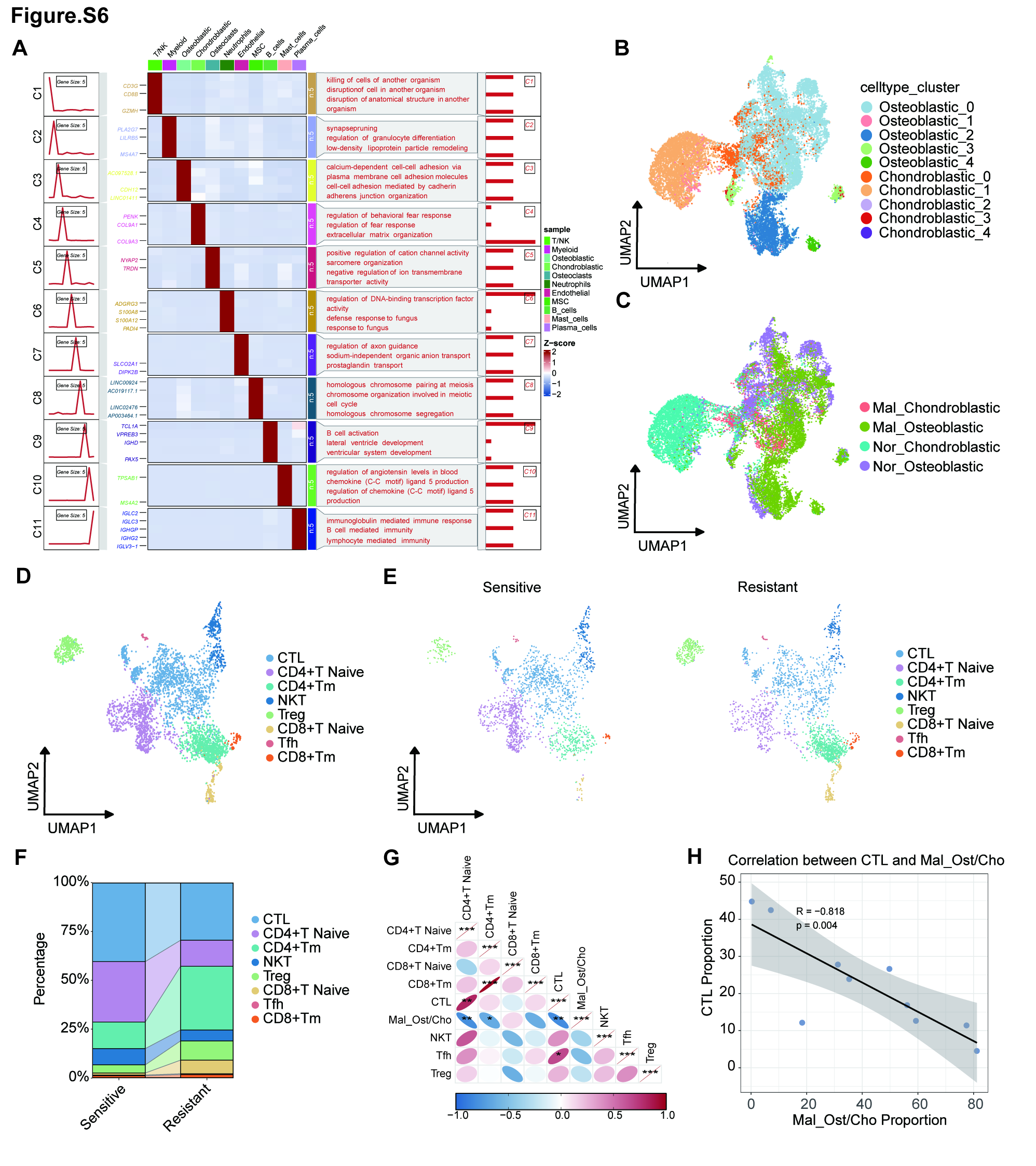

Supplement: Supplementary Figure 6 — Functional characterization and the interaction between malignant cells and T/NK cells in osteosarcoma. (A) Module analysis of gene expression programs across cell subsets using gene set variation and enrichment scores; top associated GO terms are shown. (B) Ost/Cho cell type composition in osteosarcoma samples. (C) UMAP plots showing distribution of Mal_Ost/Cho and Nor_Ost/Cho cells. (D) UMAP of T/NK cells in GitHub. CTLs: cytotoxic T lymphocytes. CD4+ naïve T cells. CD4+Tm: CD4+ memory T cells. NKT cells. Treg: regulatory T cells. CD8+ naïve T cells. Tfh: follicular helper T cell. CD8+Tm: CD8+ memory T cells. (E) T/NK cell subtypes distribution in chemo-sensitive and chemo-resistant tissues. (F) T/NK cell subtypes (GitHub) frequencies across normal and PT groups. (G) Correlation heatmap between Mal_Ost/Cho and T/NK cell subpopulations. The values are displayed on a color scale from blue (negative correlation) to red (positive correlation), with significant correlations marked by asterisks. (H) Pearson correlation analysis of CTL and Mal_Ost/Cho proportion. *P < 0.05, **P < 0.01, ***P < 0.001. [file Image6.tif]

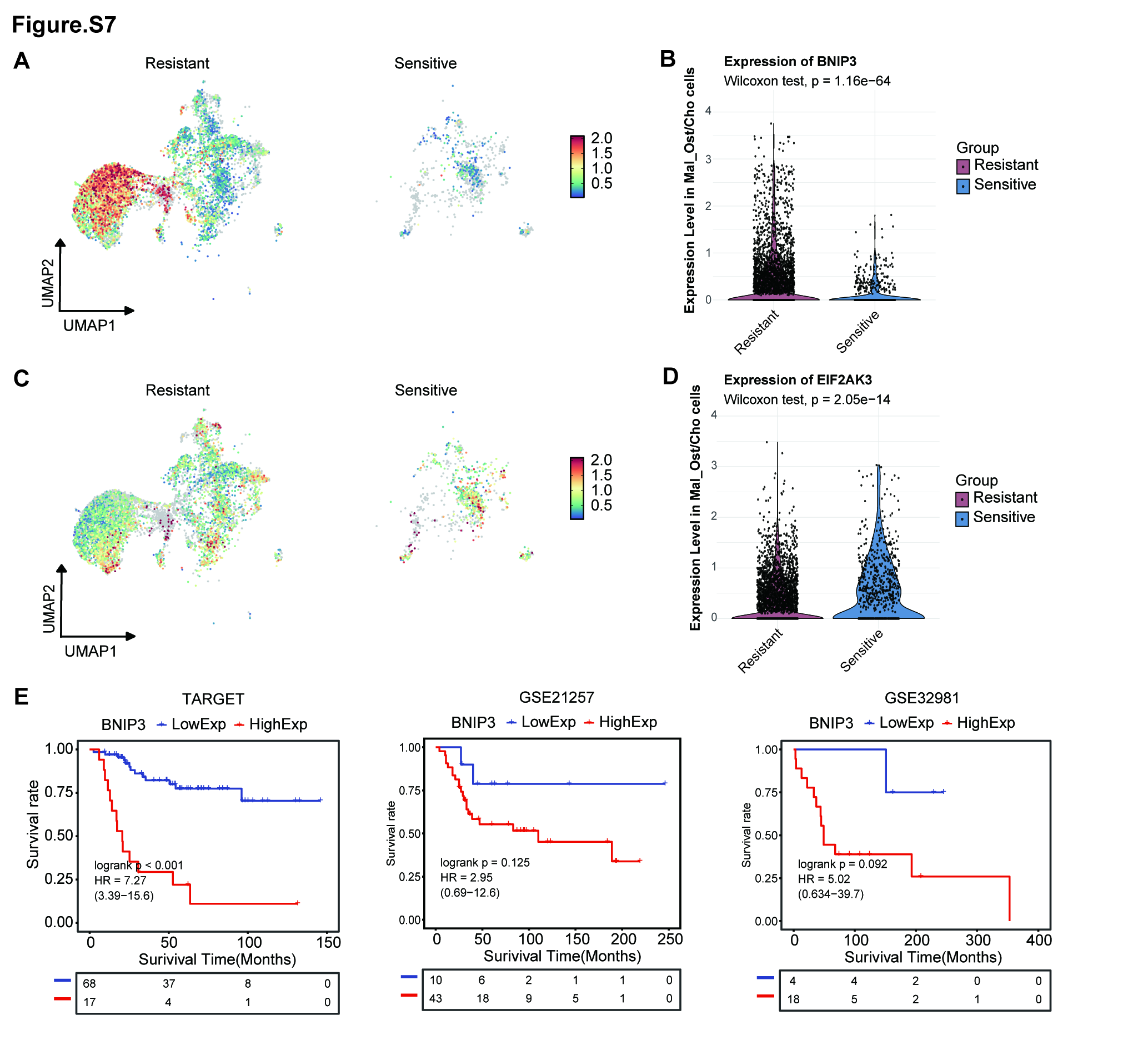

Supplement: Supplementary Figure 7 — Clinical relevance of key genes in osteosarcoma. (A) BNIP3 expression pattern in Ost/Cho subpopulations among chemo-resistant and chemo-sensitive groups. (B) Expression levels of BNIP3 in malignant Ost/Cho subtypes among resistant and sensitive groups. (C) EIF2AK3 expression pattern in Ost/Cho subpopulations among chemo-resistant and chemo-sensitive groups. (D) Expression levels of EIF2AK3 in malignant Ost/Cho subtypes among resistant and sensitive groups. (E) Kaplan-Meier survival analysis of BNIP3 expression in the TARGET, GSE21257, and GSE32981 osteosarcoma cohorts; P-values by log-rank test and HR. [file Image7.tif]

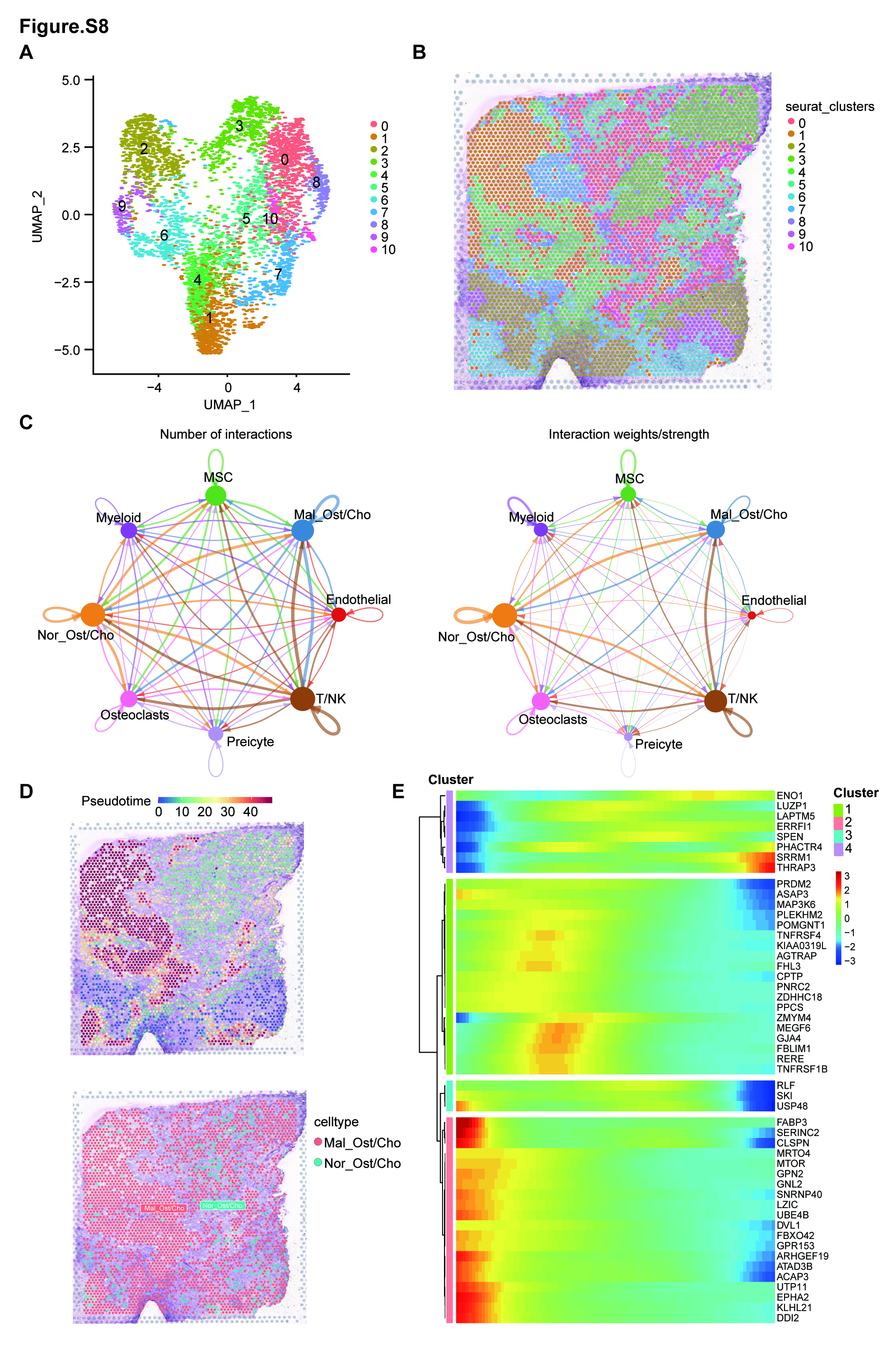

Supplement: Supplementary Figure 8 — Spatial transcriptomic analysis reveals metabolic features and intercellular communication in osteosarcoma. (A, B) UMAP plot (A) and spatial mapping (B) of cell clusters identified using Seurat (clusters 0-10). (C) Network diagrams showing the number (left) and strength (right) of predicted ligand-receptor interactions among major cell populations in the spatial transcriptomic dataset. (D) Spatial visualization of pseudotime (top) and malignant vs. normal Ost/Cho cells distribution (bottom). (E) Branched heatmap showing expression dynamics of key genes across pseudotime-associated clusters. [file Image8.tif]

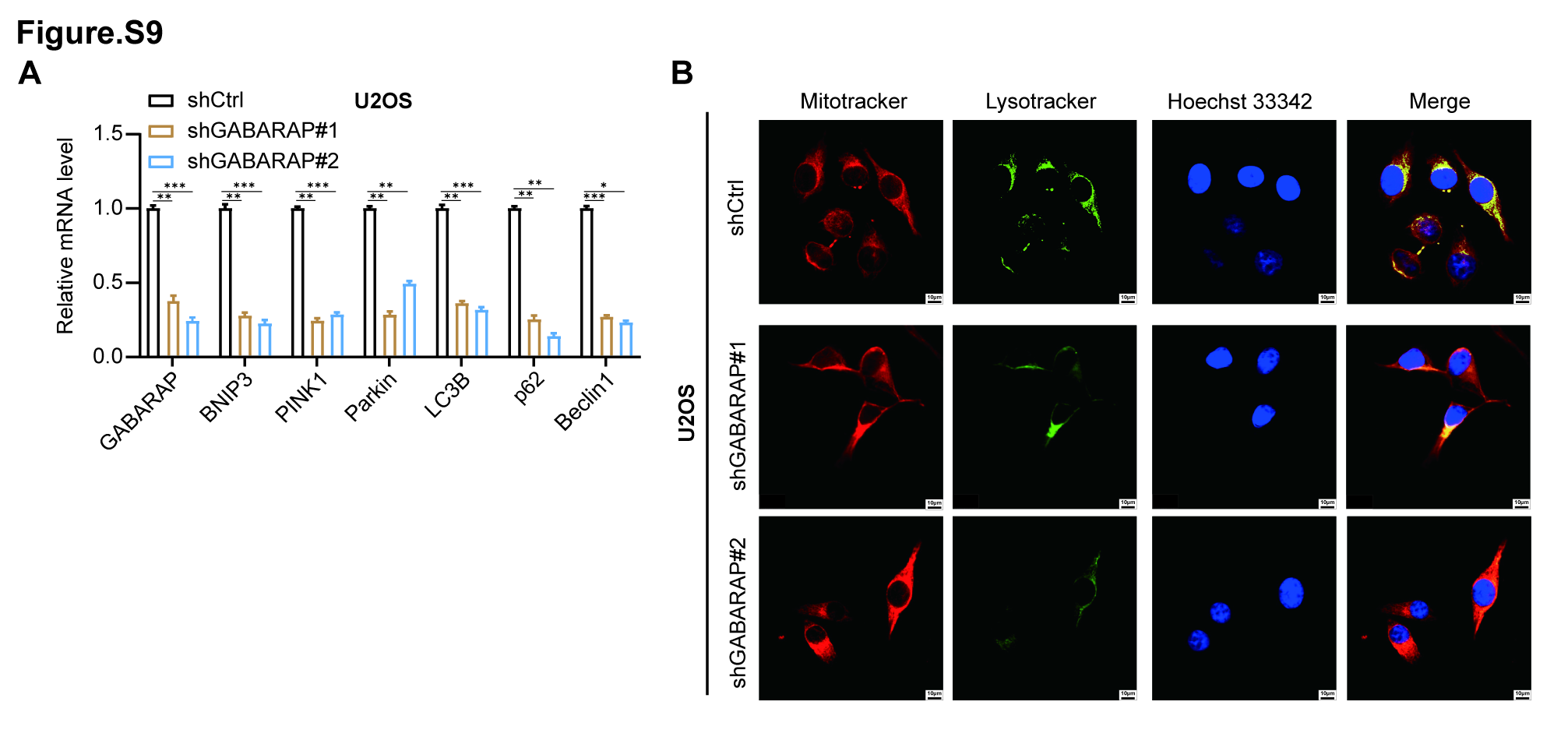

Supplement: Supplementary Figure 9 — GABARAP knockdown impairs mitophagy and mitophagy-related gene expression in U2OS cells. (A) qRT-PCR analysis of GABARAP, BNIP3, PINK1, Parkin, LC3B, p62 and Beclin1 mRNA levels in U2OS cells after GABARAP knockdown. (B) Confocal microscopy images of U2OS cells labeled with MitoTracker (red), LysoTracker (green), and Hoechst 33342 (blue). Merged images show reduced colocalization of mitochondria and lysosomes in GABARAP-silenced cells (shGABARAP#1 and shGABARAP#2). Data are shown as mean ± s.d. from three independent experiments. *P < 0.05, **P < 0.01, ***P < 0.001. [file Image9.tif]
