# Supplementary material for: Integrated multi-omics reveals GABARAP-mediated mitophagy and pyruvate metabolism as key drivers of osteosarcoma progression
Source: Front Immunol. 2025 Nov 19;16:1680554. doi: 10.3389/fimmu.2025.1680554 (PMC12672450; doi:10.3389/fimmu.2025.1680554)
Supplement: Supplementary file 13 [file Table4.docx]

**Supplementary material and methods**

**Quantitative Real-Time PCR (qRT-PCR)**

Total RNA was extracted using TRIzol reagent (Yeasen, 19201ES60, Shanghai, China) and reverse transcribed into cDNA using a commercial cDNA synthesis kit (Yeasen, 12946ES24, Shanghai, China). Quantitative PCR was performed using the SYBR Green PCR Kit (Yeasen, 11143ES50, Shanghai, China) on a CFX Connect Real-Time PCR System (Bio-Rad, CA, USA). Gene expression levels were normalized to GAPDH and calculated using the 2^-^ΔΔCT method. Primer sequences are listed in Supplementary Table S2.

**Western Blotting**

Total cellular proteins were extracted using RIPA buffer supplemented with protease (PMSF) and phosphatase inhibitors (Solarbio, R0020, Beijing, China). Protein lysates were denatured at 100 °C for 10 minutes with 5× SDS sample buffer. Equal amounts of protein were separated via SDS-PAGE and transferred to PVDF membranes (Millipore, Millipore, USA). Membranes were blocked with 8% non-fat milk (Biosharp, BS102-500g, Anhui, China) for 2 hours at room temperature and incubated overnight at 4 °C with primary antibodies against GABARAP (1:1000; Proteintech, 18723-1-AP, China) or β-actin (1:12000; Affinity, AF7018, China). HRP-conjugated secondary antibodies were applied for 1 hour at 37 °C. Finally, protein bands were visualized using ECL western blotting reagents (Yeasen, 36208ES60, Shanghai, China) and an imaging system (Bio-Rad, USA).

**Plasmid Construction and Generation of Stable Cell Lines**

Short hairpin RNA (shRNA) sequences targeting GABARAP were synthesized (TSINGKE, Beijing, China) and cloned into the pLKO.1 lentiviral vector (TSINGKE, Beijing, China). The constructs were validated via Sanger sequencing. Plasmid transfection into packaging cells was carried out using polyethyleneimine (PEI; Yeasen, Shanghai, China). The sequences of GABARAP shRNAs were: CACCATGAAGAAGACTTCTTT and GTGCCTTCTGATCTCACAGTT.
